# Long-term enriched methanogenic communities from thermokarst lake sediments show species-specific responses to warming

**DOI:** 10.1093/femsmc/xtaa008

**Published:** 2020-10-24

**Authors:** Michiel H in 't Zandt, Jeroen Frank, Polen Yilmaz, Geert Cremers, Mike S M Jetten, Cornelia U Welte

**Affiliations:** Department of Microbiology, Institute for Water and Wetland Research, Radboud University, Heyendaalseweg 135, 6525 AJ Nijmegen, the Netherlands; Netherlands Earth System Science Centre, Utrecht University, Heidelberglaan 2, 3584 CS Utrecht, the Netherlands; Department of Microbiology, Institute for Water and Wetland Research, Radboud University, Heyendaalseweg 135, 6525 AJ Nijmegen, the Netherlands; Soehngen Institute of Anaerobic Microbiology, Radboud University, Heyendaalseweg 135, 6525 AJ Nijmegen, the Netherlands; Department of Microbiology, Institute for Water and Wetland Research, Radboud University, Heyendaalseweg 135, 6525 AJ Nijmegen, the Netherlands; Department of Microbiology, Institute for Water and Wetland Research, Radboud University, Heyendaalseweg 135, 6525 AJ Nijmegen, the Netherlands; Department of Microbiology, Institute for Water and Wetland Research, Radboud University, Heyendaalseweg 135, 6525 AJ Nijmegen, the Netherlands; Netherlands Earth System Science Centre, Utrecht University, Heidelberglaan 2, 3584 CS Utrecht, the Netherlands; Soehngen Institute of Anaerobic Microbiology, Radboud University, Heyendaalseweg 135, 6525 AJ Nijmegen, the Netherlands; Department of Microbiology, Institute for Water and Wetland Research, Radboud University, Heyendaalseweg 135, 6525 AJ Nijmegen, the Netherlands; Soehngen Institute of Anaerobic Microbiology, Radboud University, Heyendaalseweg 135, 6525 AJ Nijmegen, the Netherlands

**Keywords:** methane, methanogens, permafrost, Arctic, thermokarst lakes, global warming

## Abstract

Thermokarst lakes are large potential greenhouse gas (GHG) sources in a changing Arctic. In a warming world, an increase in both organic matter availability and temperature is expected to boost methanogenesis and potentially alter the microbial community that controls GHG fluxes. These community shifts are, however, challenging to detect by resolution-limited 16S rRNA gene-based approaches. Here, we applied full metagenome sequencing on long-term thermokarst lake sediment enrichments on acetate and trimethylamine at 4°C and 10°C to unravel species-specific responses to the most likely Arctic climate change scenario. Substrate amendment was used to mimic the increased organic carbon availability upon permafrost thaw. By performing *de novo* assembly, we reconstructed five high-quality and five medium-quality metagenome-assembled genomes (MAGs) that represented 59% of the aligned metagenome reads. Seven bacterial MAGs belonged to anaerobic fermentative bacteria. Within the Archaea, the enrichment of methanogenic *Methanosaetaceae*/*Methanotrichaceae* under acetate amendment and *Methanosarcinaceae* under trimethylamine (TMA) amendment was not unexpected. Surprisingly, we observed temperature-specific methanogenic (sub)species responses with TMA amendment. These highlighted distinct and potentially functional climate-induced shifts could not be revealed with 16S rRNA gene-based analyses. Unraveling these temperature- and nutrient-controlled species-level responses is essential to better comprehend the mechanisms that underlie GHG production from Arctic lakes in a warming world.

## Teaser

Methane-cycling microorganisms of Arctic thaw lake sediments from Alaska showed species-specific temperature responses to a long-term Arctic warming scenario that was combined with substrate amendment to mimic the thaw-induced release of organic carbon.

## INTRODUCTION

Thermokarst lakes, which are widespread in the Arctic and subarctic landscape, are important greenhouse gas (GHG) sources in a warming world (Osterkamp *et al*. 2009; Deshpande *et al*. 2015; Schuur *et al*. 2015; Matveev *et al*. 2016). Methane (CH_4_) emissions are of special interest due to their strong global warming potential of 34 for 100 years (compared with the climate impact of the same quantity of CO_2_) (Myhre *et al*. 2013). It is important to consider the time horizon because of the relatively short lifetime of CH_4_ in the atmosphere. Currently, thermokarst lakes release an estimated 4.1 ± 2.2 Tg CH_4_ y^−1^, which equals 2.2% of global wetland CH_4_ emissions (Saunois *et al*. 2016; Wik *et al*. 2016). In a warming world, however, both the increase in organic matter bioavailability and elevated temperatures can induce microbial respiration, resulting in rapid oxygen depletion, production of intermediates and subsequent stimulation of methanogenesis in these lakes (van Huissteden *et al*. 2011; Deshpande *et al*. 2017; Dean *et al*. 2018).

Several studies indicate that CH_4_ production in high-latitude wetlands is mainly limited by substrate availability and, to a lesser extent, by low temperatures (Valentine, Holland and Schimel 1994; Hershey, Northington and Whalen 2014; Matheus Carnevali *et al*. 2015; de Jong *et al*. 2018; Chang *et al*. 2019). With warming-induced thaw progression, the release of labile organic matter is expected to increase (Ewing *et al*. 2015; Mueller *et al*. 2015). Under anoxic conditions, this has the potential to increase CH_4_ emissions from thermokarst lakes. However, experimental data on the link between warming, increased substrate availability and CH_4_ production in these lake sediments are not well explored.

In our previous study we performed combined experimental warming and substrate amendments on thermokarst lake sediments from Utqiaġvik, Alaska, to mimic the expected warming-induced increase of *in situ* substrates (de Jong *et al*. 2018). Amendments were performed in separate triplicate incubations to investigate the substrate-specific responses of the microbial community. The increase in temperature from 4°C to 10°C reduced methanogenic lag phases and increased methanogenesis rates with up to 30% for acetate (acetoclastic methanogenesis) and 38% for trimethylamine (TMA; methylotrophic methanogenesis). Hydrogenotrophic methanogenesis did not seem to play a major role in this ecosystem (3–5% conversion efficiency to CH_4_). Total CH_4_ production was not affected by temperature, which indicated that substrate availability was the main controlling factor.

It is, however, still largely unknown whether and how the prolonged exposure to warming under an increased substrate availability scenario that mimics permafrost thaw affects the species composition and the functional potential of these methanogenic communities. A first attempt to elucidate community-structure changes was made in our previous study by using a 16S rRNA gene-based approach (de Jong *et al*. 2018). Acetate amendment resulted in an increase in *Methanosaetaceae/Methanotrichaceae*; TMA amendment led to an enrichment of *Methanosarcinaceae* and *Methanosaetaceae/Methanotrichaceae*. This dataset can, however, not provide in-depth insights into changes in both species composition and metabolic potential of the microbial communities exposed to the combined warming and substrate amendment scenario.

To address the above questions, we applied full metagenome sequencing on these communities to unravel species-specific responses to the applied climate change scenario. With the full metagenome datasets, we could study species-level shifts that are difficult to uncover by lower resolution 16S rRNA gene-based sequencing. Understanding species-level responses is important to better comprehend the mechanisms that underlie GHG production from Arctic lakes in a warming world.

## MATERIALS AND METHODS

### Sampling site

Sediment cores were collected from two thermokarst lakes (‘Lake Emaiksoun’ and ‘Unnamed Lake’) during a winter field campaign carried out by the Vrije Universiteit Amsterdam in November 2015 to the northernmost US settlement of Utqiaġvik in the North Slope of Alaska, USA. For detailed sampling site description, see our previous study (de Jong *et al*. 2018).

### Incubations

Sediment cores were stored at 4°C until further processing. The incubation experiments were started within half a year of sampling. The elemental data from pore water data analysis were used for medium design. For medium description and incubation conditions, see de Jong *et al*. (2018). In short, triplicate incubations were prepared for acetoclastic and methylotrophic methanogens and for biotic controls without any substrate amendment. All sediment slurries had a volume of 50 mL. Samples were incubated at 4°C and 10°C. Substrates were replenished when CH_4_ production leveled off. For acetate-amended cultures, a total of 14 mM acetate was added (pulses of 4 mM at day 64 and 2 mM at days 0, 141, 190, 211 and 234) and 12 mM of TMA (pulses of 2 mM at days 0, 141, 190, 211, 234 and 269) during the 279 days of incubation.

### DNA isolation

Sediment samples were taken aseptically and pelleted by centrifugation for 10 min at 20 000 × *g*. Pellets were stored at −18°C until DNA isolation. Samples for DNA analysis were obtained by pooling equal amounts of pelleted slurry sample of each triplicate incubation. Each single DNA sample therefore represents a triplicate incubation. DNA was extracted in duplicate per pooled sample using two different extraction methods. For the first method, DNA was extracted using the PowerSoil DNA Isolation Kit (MO BIO, Qiagen, Venlo, The Netherlands) following the manufacturer's instructions with the following modifications. PowerBead Tubes were inserted in a TissueLyser LT (Qiagen, Venlo, The Netherlands) at 50 Hz. DNA was eluted from the spin column from the PowerSoil DNA Isolation Kit in two elution steps with 2 × 25 µL sterile Milli-Q. DNA samples were stored at −18°C until further analysis. For the second method, DNA was extracted using the cetyltrimethylammonium bromide (CTAB) extraction buffer protocol as described by Zhou, Bruns and Tiedje (1996). For the final step, DNA pellets were resuspended in 40 µL sterile Diethyl pyrocarbonate (DEPC)-treated Milli-Q (Invitrogen, Carlsbad, CA) by pipetting and incubation overnight at 4°C. NanoDrop analysis indicated contamination of DNA samples with organics. DNA samples were purified using Agencourt AMPure XP beads following the manufacturer's instructions (Beckman Coulter, Brea, CA). DNA quality was checked by agarose gel electrophoresis and spectrophotometrically using the NanoDrop 1000 (Invitrogen, Thermo Fisher, Carlsbad, CA). DNA quantity was measured fluorometrically by using the Qubit dsDNA HS Assay Kit (Invitrogen, Thermo Fisher, Carlsbad, CA) according to the manufacturer's instructions. The two DNA extraction methods were used to improve the resolution for downstream metagenome-assembled genome (MAG) reconstruction. In total, 12 DNA samples were prepared for metagenome sequencing.

### Metagenome sequencing

Library preparation of the metagenomes (one library per DNA extraction for each metagenome) was done using the Nextera XT kit (Illumina, San Diego, CA) according to the manufacturer's instructions. Enzymatic tagmentation was performed with 1 ng input DNA, followed by incorporation of the indexed adapters and amplification of the library. After purification of the amplified library using AMPure XP beads (Beckman Coulter, Indianapolis, IN), libraries were checked for quality and size distribution using the Agilent 2100 Bioanalyzer (Agilent, Santa Clara, CA) and the Qubit dsDNA HS Assay Kit. Quantification of the library was performed with the Qubit dsDNA HS Assay Kit. The libraries were pooled, denatured and sequenced with the Illumina MiSeq system (Illumina, San Diego, CA). Paired-end sequencing of 2 × 301 base pairs was performed using the MiSeq Reagent Kit v3 (Illumina, San Diego, CA) according to the manufacturer's protocol.

### Metagenome analysis

Metagenome datasets were processed as previously described up to the generation of MAGs (in ’t Zandt *et al*. 2019). Taxonomic assignments for MAGs were based on the Genome Taxonomy Database with the GTDB-Tk tool v0.2.1 (Chaumeil *et al*. 2019). Reads containing parts of the 16S ribosomal RNA (rRNA) gene were identified by performing a BLASTN search of the quality-filtered reads to the SILVA SSU Ref NR 99 release 132 database using a length and similarity fraction of 50% and 70%, respectively (Quast *et al*. 2012). Mapping was done in the CLC Genomics Workbench 11.0 using the BLASTN algorithm with default settings (CLCbio, Aarhus, Denmark). Mapped reads were size filtered for a minimum length of 200 base pairs. Reads were submitted to the SILVAngs pipeline and processed with the default settings for Illumina MiSeq reads. SILVAngs is an automated data analysis service for 16S rRNA gene amplicon reads from high-throughput sequencing. It uses the SILVA 16S rRNA gene databases, taxonomies and alignments as a reference. Taxonomic frequencies were exported and used for the analysis of the taxonomic composition of the microbial samples. The bins were gene-called by Prodigal and annotated (cutoff = E−50) using a custom hidden markov model (HMM) database and HMMER (http://hmmer.org/) (Eddy 2011). To build this database, proteins from the TrEMBL database of EMBL were selected based on their presence in the Kyoto Encyclopedia of Genes and Genomes (KEGG) metabolic pathways and clustered using Linclust(--kmer-per-seq 160 --min-seq-id 0.5 --similarity-type 1 --sub-mat blosum80 --cluster-mode 2 --cov-mode 0 -c 0.7). The clusters were subsequently aligned with mafft() (--anysymbol) and HMM profiles were created with hmmbuild (default) for each cluster. Proteins of special interest for this study, like methyl-coenzyme M reductase (Mcr), were manually curated and complemented with HMMs of phylogenetic groups of interest [see Supporting Information of Poghosyan *et al*. (2020) for an overview].

For functional gene searches, Prokka RefSeq-annotated MAGs were imported in Artemis v17 (Rutherford *et al*. 2000; Seemann 2014). We used the RefSeq database to provide more accurate annotations of especially archaeal MAGs. Here, we focused on genes that were relevant for carbon, methane and additionally key nitrogen cycling genes. For retrieval of heterodisulfide reductase (HdrDE) sequences, reviewed Swiss-Prot HdrDE sequences from *Methanosarcina acetivorans*, *M. barkeri*, *M. mazei* and *M. thermophila* were downloaded from the UniProt Knowledgebase (UniProtKB) on 9 April 2020 (The UniProt Consortium 2014). The annotated *Methanosarcina* MAGs were blasted against the HdrDE database using BLASTP to retrieve HdrDE hits. All target genes were blasted against the NCBI non-redundant protein database using the BLASTP algorithm. Gene clusters represent multiple genes that are present as clusters in prokaryotic genomes.

Genome comparisons were performed by using OrthoVenn2 with default settings and the ANI/AAI-Matrix genome-based distance matrix calculator (Rodriguez-R and Konstantinidis 2016; Xu *et al*. 2019).

## RESULTS AND DISCUSSION

**Figure 1.**
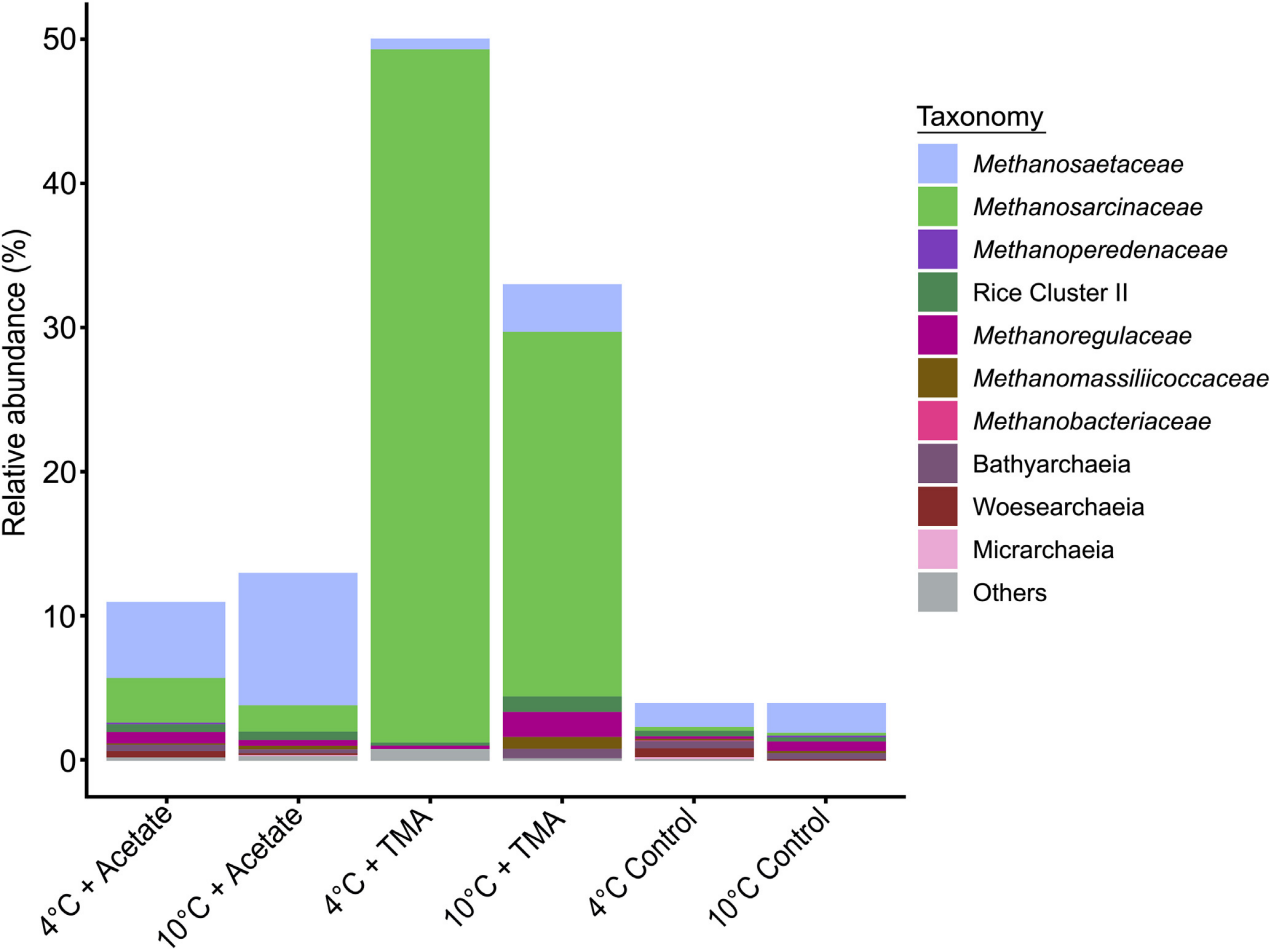
Taxonomic distribution of archaeal 16S rRNA gene reads obtained from metagenomic datasets of methanogenic incubations with acetate, TMA and control at 4°C and 10°C. A total of 0.12% of reads were identified as 16S rRNA gene-containing sequences. The group ‘Others’ includes all taxonomic groups with a relative abundance <1% within the sample. Taxonomic identification is given up to family level. The *Y*-axis displays the relative abundance with 100% being the sum of metagenome-derived archaeal and bacterial 16S rRNA gene reads per sample, including non-classified reads (≤5%).

Here, we used metagenomics to unravel community- and species-specific responses to the most likely Arctic climate change scenario of a 6°C increase in temperature by 2100. A total of 12 libraries were sequenced. The average library depth was 5.2 million reads with a sequence quality score >Q_30_ for 73.5% of the reads. Metagenome-derived 16S rRNA gene reads were analyzed to gain insights into relative abundance shifts in the microbial communities with and without substrate amendment under the two climate scenarios. The observed changes within the methanogenic communities were in line with the previous observations and showed an enrichment of methanogens on both acetate and TMA incubations (Fig. 1; see Table S1, Supporting Information). The strongest enrichment in methanogenic archaea was observed upon TMA amendment.

**Figure 2.**
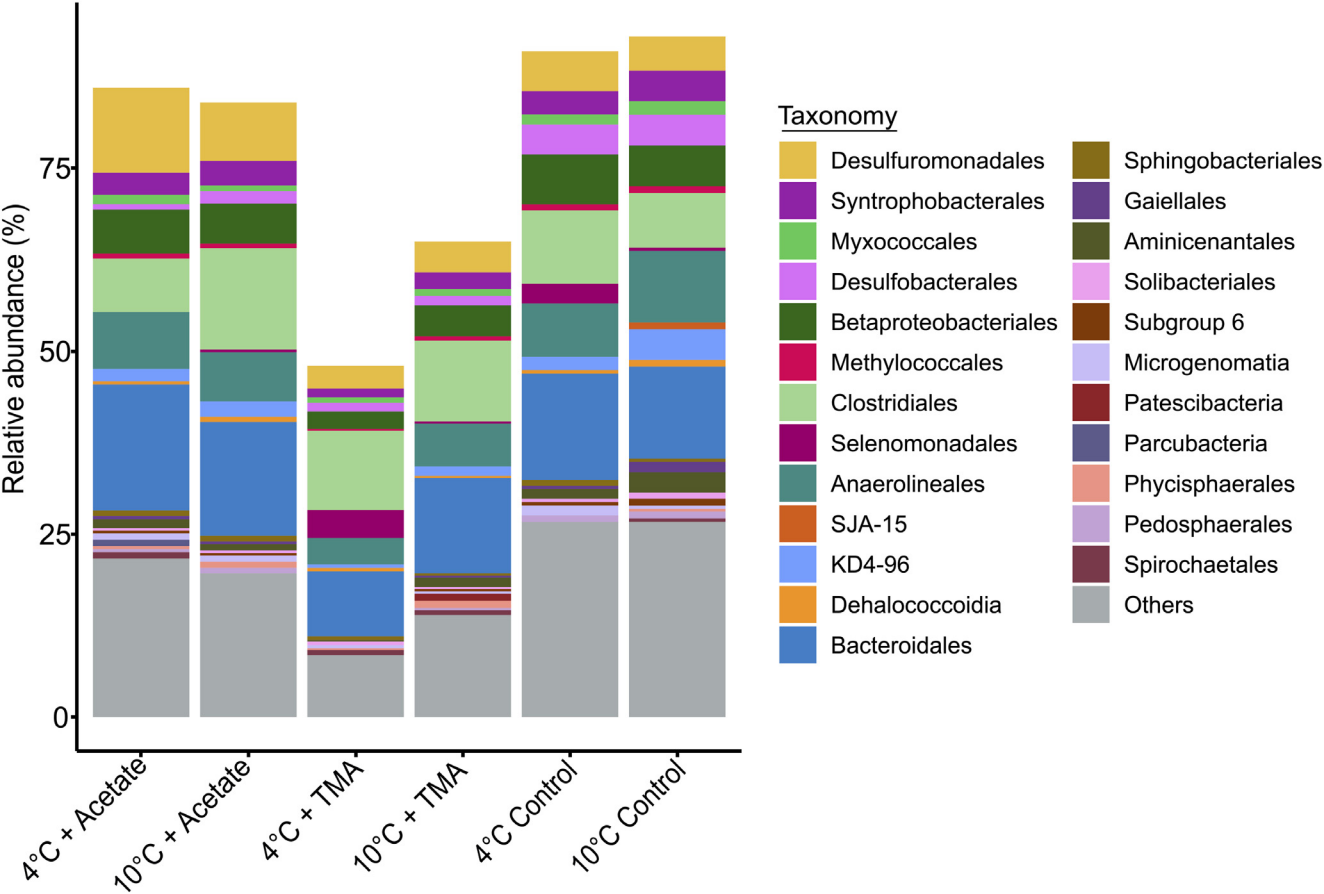
Taxonomic distribution of bacterial 16S rRNA gene reads obtained from metagenomic datasets from methanogenic incubations with acetate, TMA and control incubations at 4°C and 10°C. A total of 0.12% of reads were identified as 16S rRNA gene-containing sequences. All taxonomic groups have a relative abundance of ≥1%. The group ‘Others’ includes all taxonomic groups with a relative abundance <1%. Taxonomic identification is given up to order level. The *Y*-axis displays the relative abundance with 100% being the sum of metagenome-derived archaeal and bacterial 16S rRNA gene reads per sample, including non-classified reads (≤5%).

The pronounced community differences in the substrate-amended incubations were caused by substrate-specific responses of the methanogenic population (Conrad 2002). Acetoclastic *Methanosaetaceae/Methanotrichaceae* were mainly enriched on acetate, with a 1.7-fold stronger response at 10°C. On TMA, versatile *Methanosarcinaceae* showed strongest enrichment of up to 48% of the total prokaryotic community, with a 1.9-fold higher enrichment at 4°C. In contrast, the bacterial community showed less pronounced responses (Fig. 2; see Table S2, Supporting Information).

Overall, the bacterial communities were highly diverse and did not show pronounced differences between the substrate amendments and the two temperatures. All communities were dominated by Desulfuromonadales, Betaproteobacteriales, Clostridiales, Anaerolineales and Bacteroidales. In our previous 16S rRNA gene-based study, we also observed a dominance of Desulfuromonadales, Clostridiales and Bacteroidales in the incubations on acetate and TMA; Anaerolineales were abundant in all incubations and Betaproteobacteriales were below the detection limit (<2% relative 16S rRNA gene read abundance) (Fig. S4 of de Jong *et al*. 2018). On acetate, the clearest responses were a temperature-specific decrease of Desulfuromonadales (11.6% relative abundance at 4°C, and 8.0% relative abundance at 10°C) and an increase in Clostridiales (7.3% relative abundance at 4°C, and 13.9% relative abundance at 10°C) 16S rRNA gene reads upon the temperature shift. Upon TMA amendment, the most pronounced shifts were an increase in Betaproteobacteriales (2.4% relative abundance at 4°C, and 4.2% relative abundance at 10°C), Anaerolineales (3.6% relative abundance at 4°C, and 5.9% relative abundance at 10°C) and Bacteroidales (8.9% relative abundance at 4°C, and 13.0% relative abundance at 10°C). However, these differences were only observed between single samples, and more in-depth analyses and replications are needed to statistically confirm our observations. In addition, short 16S rRNA gene-containing DNA sequences have a limited resolution for taxonomic identification.

To obtain more insights into the species-specific changes upon methanogenic substrate amendment and temperature increase, we applied *de novo* assembly and consensus binning to construct MAGs. Binning resulted in 58 MAGs, including five high-quality drafts (>90% completeness, <5% redundancy) and five medium-quality drafts (>70% completeness, <10% redundancy) (see Table S3, Supporting Information) (Bowers *et al*. 2017). In total, 77% of the reads could be aligned to all MAGs, with 59% of the reads aligning to the 10 most complete MAGs (Table 1). Three of the top 10 MAGs were identified as methanogenic *Methanosarcinaceae*. The sequencing coverage of the MAGs was assessed to obtain insights in their relative abundance under the different nutrient amendment and temperature scenarios.

**Table 1.** Abundance for the 10 MAGs with >70% completeness. Taxonomic identity was assessed by the GTDB-Tk toolkit. Completeness was assessed by CheckM. Abundance percentages were obtained by mapping back the quality-filtered reads per sample to the MAGs and by averaging the total read corrected coverage data per DNA extraction method. MAG: metagenome-assembled genome, TMA: trimethylamine.

#	Taxonomy	Completeness (%)	4°C acetate	10°C acetate	4°C TMA	10°C TMA	4°C control	10°C control
MAG 1	*Pelobacteraceae* (Family)	100.0	20.2%	20.1%	1.9%	3.2%	8.6%	5.0%
MAG 2	Bacteroidales (Order)	99.5	3.3%	3.0%	4.3%	1.8%	4.7%	1.7%
MAG 3	Peptostreptococcales (Order)	99.3	5.2%	7.7%	3.3%	4.4%	10.1%	2.1%
MAG 4	*Methanosarcinaceae* (Family)	97.2	0.4%	0.1%	52.2%	11.5%	0.0%	0.0%
MAG 5	*Methanosarcinaceae* (Family)	96.8	0.0%	0.0%	0.2%	26.8%	0.0%	0.0%
MAG 6	Bacteroidales vadinHA17 (Order)	96.6	5.1%	3.7%	1.2%	1.8%	3.8%	3.6%
MAG 7	Elusimicrobiales (Order)	88.8	1.8%	0.9%	0.4%	0.9%	5.8%	5.4%
MAG 8	Elusimicrobiales (Order)	86.0	0.1%	1.6%	0.0%	1.4%	4.1%	6.3%
MAG 9	*Methanosarcinaceae* (Family)	84.5	8.6%	4.0%	0.6%	10.3%	0.2%	0.1%
MAG 10	Anaerolineales (Order)	77.6	2.1%	2.2%	0.5%	1.4%	3.2%	4.0%

### Fermenting bacterial groups dominate the nutrient-amended communities

The anaerobic incubations both with and without substrate additions contained a high amount of anaerobic fermentative bacteria, which constituted the majority of the microbial communities and were also dominant in the original cores (Garrity, Bell and Lilburn 2004) (Table 1; see Table S7, Supporting Information, and Fig. 1 of de Jong *et al*. 2018). Binning resulted in the reconstruction of seven bacterial MAGs: one MAG was identified as *Pelobacteraceae* (Order: Desulfuromonadales), two MAGs were identified as Bacteroidales, two MAGs as Elusimicrobiales, one as Anaerolineales and one MAG was identified as Peptostreptococcales (Class: Clostridia) (Table 1). The MAG identification was supported by 16S/23S rRNA gene and RpoB (DNA-directed RNA polymerase subunit B) identities.

The Desulfuromonadales/*Pelobacteraceae* MAG showed a strong response on acetate amendment but not to the different temperatures (Table 1). It has been shown for several *Pelobacter* species that acetate is required as carbon source for growth, but that it is not used in central energy metabolism (Schink 1985; Lovley *et al*. 1995; Richter *et al*. 2007). The MAG reported here contains several genes encoding cation acetate symporters and an acetate kinase (see Table S7, Supporting Information). The increase in coverage of the *Pelobacteraceae* MAG upon acetate amendment indicates the supportive role of acetate for growth. The absence of a temperature response in the *Pelobacteraceae* MAG indicates a potentially psychrophilic nature of the species.

The central metabolism of *Pelobacteraceae* revolves around the metabolism of a wide variety of organic compounds, including complex compounds like trihydroxybenzenes and polyethyleneglycol, linked to the reduction of alternative electron acceptors (Schink and Pfennig 1982; Schink and Stieb 1983). For *Pelobacter carbinolicus*, it was reported that Fe(III) and elemental sulfur can be used as alternative electron acceptors (Lovley *et al*. 1995). In permafrost environments, Desulfuromonadales sequences have been linked to sulfur and metal reduction (Gittel *et al*. 2014; Dao *et al*. 2018). The *Pelobacteraceae* MAG described in our study contains several genes encoding *c*-type cytochromes, including a cytochrome *c*3 that is potentially involved in metal reduction (Lovley *et al*. 1995). However, in the long-term incubations, oxidized metals and elemental sulfur are likely rapidly depleted. Since these alternative electron acceptors were not added, they probably played only minor roles.

Specific co-occurrences with methanogenic archaea have only been described in a few studies. The early work by Bryant and co-workers describes a culture of ‘*Methanobacillus omelianskii*’, a co-culture of *Pelobacter* with hydrogenotrophic methanogens (Bryant *et al*. 1967). A study by Timmers and co-workers on methanogenic, sulfate-reducing sludge found a co-existence of methanogens and *Pelobacter*-related Desulfuromonadales (Timmers *et al*. 2015). Here, we also observed their co-occurrence with methanogenic archaea, more specifically with *Methanosarcinaceae*, in both substrate-amended and -unamended sediments that highlight their potential methanogen-supporting role in organic-rich methanogenic ecosystems.

The two Bacteroidales MAGs were present in all conditions (Table 1). Bacteroidales are dominant players in organic-rich lake sediments where they can play an important role in polysaccharide degradation (Schwarz, Eckert and Conrad 2007; Thomas *et al*. 2011; He *et al*. 2015; Wang *et al*. 2016). A 16S rRNA gene amplicon study by Wang and co-workers detected Bacteroidetes among the key players in soil and lake sediments from London Island, Svalbard (Wang *et al*. 2016). In addition, a study on the CH_4_ food web in Arctic sediments found Bacteroidetes among the dominant microorganisms, based on 16S rRNA gene pyrosequencing data (He *et al*. 2015). Our observations are in line with previous studies and highlight their potential role in supporting methanogenic communities. Interestingly, only minor responses to substrate amendment were observed, which indicates that the two Bacteroidales MAGs are probably mainly controlled by the availability of *in situ* polymeric substrates. The observation that Bacteroidales were more abundant in the original cores supports these observations (Fig. 1 of de Jong *et al*. 2018).

The Peptostreptococcales MAG was present in all samples and showed a minor response to substrate amendment (Table 1). Peptostreptococcales/Clostridia possess a wide array of fermentation pathways and are common inhabitants of soils and sediments (Tracy *et al*. 2012). They are common inhabitants of permafrost soils and sediments, where they can play important roles in the production of methanogenic substrates, including acetate, formate and H_2_ (Lipson *et al*. 2013; Tveit *et al*. 2015; Heslop *et al*. 2019). Specifically for *Peptostreptococcaceae*, their fermentative metabolism is often linked to acetate production (Slobodkin 2014). The Peptostreptococcales MAG described here contained genes encoding acetate kinase and acetyl-CoA synthase (see Table S7, Supporting Information). Several members of the Clostridia have been linked to the degradation of cellulose and humic substances (Lynd *et al*. 2002; Ueno *et al*. 2016), but this metabolic potential was not detected in the Peptostreptococcales MAG.

The 10 most abundant MAGs included three MAGs of the less well-studied orders Elusimicrobiales and Anaerolineales. The phylum Elusimicrobia (formerly ‘Termite Group 1’) is widespread in soils and sediments, including Arctic lakes (Herlemann, Geissinger and Brune 2007; Negandhi, Laurion and Lovejoy 2016; Wang *et al*. 2016). However, little is known about their role in the environment (Brune 2018). A recent study by Méheust *et al*. (2020) on groundwater Elusimicrobia highlighted their potential in nitrogen cycling by the detection of a nitrogenase paralog in several MAGs. In our study, two MAGs were identified as Elusimicrobiales (Table 1). The MAG identifications were supported by 16S/23S and RpoB analyses that showed highest identity to *Elusimicrobia*-related sequences (data not shown). Both MAGs showed highest abundances in the unamended sediments (9.0–9.9%) but covered <2.5% of the nutrient-amended communities. Interestingly, both MAGs contained the *nrfA* gene encoding nitrite reductase (see Table S8, Supporting Information). Furthermore, the MAGs contained acetate and butyrate kinases, which indicates a fermentative lifestyle (see Table S7, Supporting Information). Interestingly, our observations highlight that increased nutrient availability reduces their relative abundance. Further experimental evidence is needed to confirm their role in the environment.

We also reconstructed an Anaerolineales MAG with highest coverages in the unamended sediments (Table 1). The class Anaerolineae consists of chemoorganotrophic bacteria within the phylum Chloroflexi (Yamada *et al*. 2006). Our previous 16S rRNA gene-based dataset highlighted the presence of Anaerolineales in the bacterial communities of the original sediment cores (4–8% relative abundance) (de Jong *et al*. 2018). The reconstructed MAG was highly fragmented and contained eight 16S rRNA gene sequences that did not support further species identification. Closer identification on Rpo sequences indicated highest identity to environmental sequences of *Anaerolineaceae* (data not shown). The genome contained NADH dehydrogenase (*nuoABDEFGHIJKLMN*), succinate dehydrogenase (including the cytochrome *b*_556_ subunit) and cytochrome *d* ubiquinol oxidase subunit I and II that are part of the aerobic respiratory chain. A cytochrome *c* nitrite reductase subunit *c*_552_ and a nitric oxide reductase link to its potential to use nitrate/nitrite as terminal electron acceptor (Chen and Strous 2013). However, we could not detect genes involved in fermentation, nitrogen and sulfur metabolism (see Tables S7 and S8, Supporting Information). In addition, little data are available on their occurrence in natural ecosystems, and data on their potential metabolic role are lacking (Huang *et al*. 2019). Further research into their environmental role is therefore highly needed.

### Acetate amendment results in an increase of acetoclastic *Methanosaetaceae/Methanotrichaceae*

Acetate is a major methanogenic substrate at lower temperatures, mainly due to a reduction in syntrophic activity and an increase in homoacetogenesis (Kotsyurbenko 2005; Schulz, Matsuyama and Conrad 2006; Blake *et al*. 2015). For the thermokarst lake sediments studied here, this was supported by a dominance of strictly acetoclastic *Methanosaetaceae/Methanotrichaceae* in the unamended sediment incubations at both temperatures. *Methanosaetaceae/Methanotrichaceae* are common inhabitants of thermokarst lake sediments, including the thermokarst lake sediments studied here (Negandhi *et al*. 2013; de Jong *et al*. 2018; Matheus Carnevali *et al*. 2018). Upon acetate amendment, a further increase in *Methanosaetaceae/Methanotrichaceae* was observed (9.0% and 4.1% of the aligned reads at 4°C and 10°C, respectively). Versatile *Methanosarcinaceae* were also detected, but at lower relative abundances.

Due to their low substrate threshold *Methanosaetaceae/Methanotrichaceae* are expected to dominate over *Methanosarcinaceae* in substrate-limited conditions (Westermann, Ahring and Mah 1989). Minimum acetate threshold concentrations for *Methanosaetaceae*/*Methanothrix* sp. are below 0.01 mM, whereas *Methanosarcina* sp. have a much higher substrate limit of 0.2–1.2 mM (Westermann, Ahring and Mah 1989; Jetten, Stams and Zehnder 1990, 1992). Upon acetate amendment at low substrate concentrations (2–4 mM), we observed a clear increase of *Methanosaetaceae/Methanotrichaceae* (1.6–2.1 to 5.3–9.2%) and a lower increase in *Methanosarcinaceae* (0.2–0.3 to 1.8–3.1%). It is therefore likely that a rapid turnover of acetate, which was supported by CH_4_ production rates, led to the dominance of *Methanosaetaceae/Methanotrichaceae*.

Surprisingly, there was no acetoclastic *Methanosaetaceae/Methanotrichaceae* MAG among the dominant assembled genomes. A single *Methanosaetaceae/Methanotrichaceae* MAG could, however, be recovered with an estimated 65.5% genome completeness. This MAG showed highest read coverages on acetate at 4°C (2.0%) and 10°C (3.5%) and lower on TMA at 4°C (0.13%) and 10°C (0.30%) and unamended controls at 4°C (0.49%) and 10°C (0.61%). Low genome completion and high fragmentation of the MAG, however, hampered further analysis.

### TMA strongly induces versatile *Methanosarcinaceae* with distinct temperature responses

Upon TMA amendment, the methanogenic community was dominated by versatile *Methanosarcinaceae*. They can perform acetoclastic, methylotrophic and hydrogenotrophic methanogenesis, and they rapidly respond to increased nutrient availability (Patel and Sprott 1990; Sprenger *et al*. 2000; Spring *et al*. 2010). This is in line with the high substrate turnover efficiency of 73% that was measured in our previous study (de Jong *et al*. 2018). All three MAGs contained a partial or complete *nifDHK* gene cluster encoding the nitrogenase complex (see Table S8, Supporting Information). This nitrogen fixation potential was not observed in the other seven MAGs, indicating a potential important role of methanogenic archaea in the nitrogen cycle of this ecosystem. The three *Methanosarcinaceae* MAGs were highly abundant upon TMA amendment that covered 53.0% and 48.6% of the assigned reads at 4°C and 10°C, respectively (Table 1).

Interestingly, the MAGs showed unique responses to the temperature scenarios (Table 1). At 4°C *Methanosarcinaceae* MAG 4 dominated, whereas at 10°C all three *Methanosarcinaceae* MAGs were present, with a dominance of *Methanosarcinaceae* MAG 5. Rapid and efficient substrate conversions indicated their adaptation to low temperatures. Several *Methanosarcina* species have been isolated from cold habitats (Simankova *et al*. 2003; Morozova *et al*. 2015). Despite their growth at low temperatures (1–5°C), most isolates are psychrotolerant and show optimum growth at moderate temperatures (25–35°C) (Simankova *et al*. 2003; Wagner and Liebner 2010). *Methanosarcinaceae* MAG 5 and 9 follow this trend on TMA with increases at 10°C, indicating a preference for higher temperatures. We performed a closer investigation of the three *Methanosarcinaceae* MAGs in an attempt to gain insights into their differences. None of the methanogen MAGs contained a 16S rRNA gene. Therefore, the complete methyl-coenzyme M reductase gene cluster that was present in all three MAGs was used for species identification using protein BLASTs (the BLASTP algorithm).

*Methanosarcinaceae* MAG 4 showed a strong enrichment on TMA at 4°C and was nearly absent in the acetate-amended sediments. In MAG 4, a complete *mcrAGCDB* gene cluster was found with 91.8–99.5% average amino acid identity (AAI) to *Methanosarcina sp*. sequences. McrABG is most identical to *Methanosarcina* sp. 2.H.A.1B.4 that was obtained from a metagenome sequencing experiment on Columbia River sediment culture grown on acetate (Youngblut *et al*. 2015). Nearest cultured representatives are *Methanosarcina lacustris* (94.0–99.0% AAI for McrAGCB) and *M. acetivorans* (86.5% AAI to McrD). *Methanosarcinalacustris* is a psychrotolerant methanogen isolated from anoxic lake sediments in Switzerland (Simankova *et al*. 2001). MAG 4 contains acetate kinase (ack), phosphate acetyltransferase (pta) and acetyl-CoA synthetase (ACS) and CODH/ACS gene cluster CdhABCDE for carbon fixation through the Wood–Ljungdahl pathway.

*Methanosarcinaceae* MAG 5 showed strict enrichment on TMA and was not observed in the sediments amended with acetate. Its response showed a high-temperature specificity with a high enrichment at 10°C and low coverage at 4°C (Table 1). The *mcrAGCDB* gene cluster was most identical to *Methanosarcina* sp. (91.8–99.0% AAI). Nearest cultured representatives are *Methanosarcina spelaei* (93.7% AAI for McrA), *M. lacustris* (34.9–98.5% AAI for McrGCB) and *M. acetivorans* (85.9% AAI for McrD). *Methanosarcinaspelaei* is isolated from a floating biofilm in mesothermal water of a subsurface lake (Ganzert *et al*. 2014). Interestingly, the optimal growth temperature of *M. spelaei* is 33°C, but the organism can grow at temperatures down to 0°C (Ganzert *et al*. 2014). Its higher coverage on TMA at 10°C indicates the preference of this MAG to higher temperatures. MAG 5 contains acetate kinase (ack), phosphate acetyltransferase (pta) and acetyl-CoA synthetase (ACS) and CODH catalytic subunit (CooS, 2 copies) for carbon fixation through the Wood–Ljungdahl pathway.

*Methanosarcinaceae* MAG 9 showed strongest enrichment on acetate at 4°C (2.2-fold increase) and on TMA at 10°C (17.2-fold increase). A complete *mcrAGCDB* gene cluster was found with 97.55–100% aa identity to *Methanosarcina* sp. Ant1. Closest cultured representatives are *M. lacustris* (93.4% AAI for McrA), *M. horonobensis* (92.7% AAI for McrG, 88.5% AAI for McrB), *M. acetivorans* (96.1% AAI for McrC) and *M. barkeri* (86.0% AAI for McrD). MAG 9 contained acetate kinase (ack), phosphate acetyltransferase (pta) and acetyl-CoA synthetase (ACS) and the CODH complex.

We performed a closer functional gene and gene cluster analysis to obtain insights into the metabolic potential of the three *Methanosarcinaceae* MAGs (Table 2; see Tables S6–S8, Supporting Information). We selected up to three key genes in energy metabolism, oxidative defense mechanisms, movement, the toxin–antitoxin system and cytochromes that are reported in Methanosarcinaceae (NCBI, KEGG database).

**Table 2.** Functional gene analysis for the three Methanosarcinaceae MAGs. Green indicates presence of gene(s)/gene clusters, yellow indicates incomplete gene clusters, red indicates absence of gene(s)/gene clusters. Vho: membrane-bound F420-non-reducing hydrogenase, Hdr: heterodisulfide reductase subunits DE, Rnf: respiratory Rnf complex, cbp: cytochrome biogenesis proteins, cap: cytochrome assembly proteins, cyt c: cytochrome c proteins, TA II: type II toxin–antitoxin system, ADH: alcohol dehydrogenase, Cat: catalase, Per: peroxidase, Che: chemotaxis gene cluster, T4P: type IV pilin and Fla: flagellin.

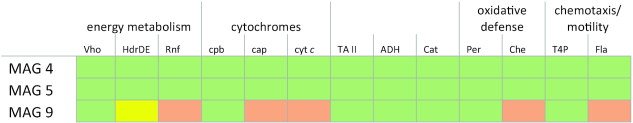

A membrane-bound F_420_-non-reducing hydrogenase (Vho) and a heterodisulfide reductase (HdrDE) were found in all *Methanosarcinaceae* MAGs. HdrDE was detected by BLASTP of *Methanosarcina* sp. sequences present in the Swiss-Prot database against the MAGs (E-values <1.69e-166) (see Table S5, Supporting Information). MAG 9 only contained HdrE. The sodium ion translocating Rnf complex that is linked to energy conservation in *Methanosarcinaceae* (Welte and Deppenmeier 2014) could not be detected in MAG 9, probably due to genome incompleteness.

All Methanosarcinales contain *c*-type cytochromes (Thauer *et al*. 2008). All MAGs contained cytochrome *c* biogenesis proteins (CcmE & CcdA) from the cytochrome *c* maturation system I, but genes encoding cytochrome *c* proteins were only found in MAG 4 and 5 (Stevens *et al*. 2011). Alcohol dehydrogenases for potential use of alcohols as electron donors were detected in all MAGs. Specific for MAG 5, an isopropanol dehydrogenase gene was identified.

All *Methanosarcinaceae* MAGs encoded genes for several toxin–antitoxin system II proteins that are proposed to provide an antiviral defense mechanism (Makarova, Wolf and Koonin 2013). The putative RNA-targeting HicAB cassette was found in all three genomes (Makarova, Grishin and Koonin 2006). The mRNA targeting interferase RelE and DNA gyrase inhibiting ParE was detected in all MAGs (Makarova, Wolf and Koonin 2009). VapC with predicted nuclease activity was not found in MAG 9; YefM that is part of the YefM-YoeB toxin–antitoxin system was only detected in MAG 5 (Cherny and Gazit 2004; Makarova, Wolf and Koonin 2009).

Catalase and peroxidase, which are involved in oxidative defense, were present in all three *Methanosarcinaceae*. All MAGs encode a NADH peroxidase. Furthermore a cytochrome *c* peroxidase detected in MAG 4 and MAG 9 additionally encodes a catalase/peroxidase HPI, a bifunctional enzyme with both catalase and broad-spectrum peroxidase activity (Hillar *et al*. 2000).

The chemotaxis gene cluster CheRDCABYW was found in MAG 4 and MAG 5, with the absence of CheY in MAG 5. The cluster is structurally similar to the one described in *M. acetivorans* and other archaea (Galagan *et al*. 2002; Schlesner *et al*. 2009). Type IV pilin and flagellar assembly proteins were found in all MAGs. However, genes encoding for flagellin were only detected in MAG 4 and MAG 5. Together with cytochrome *c* proteins, pili and flagella can play a role in direct interspecies electron transfer reactions that support a syntrophic lifestyle (Shimoyama *et al*. 2009; Shrestha *et al*. 2013). This strategy can be relevant under environmental conditions in which acetate is increasingly available.

**Figure 3.**
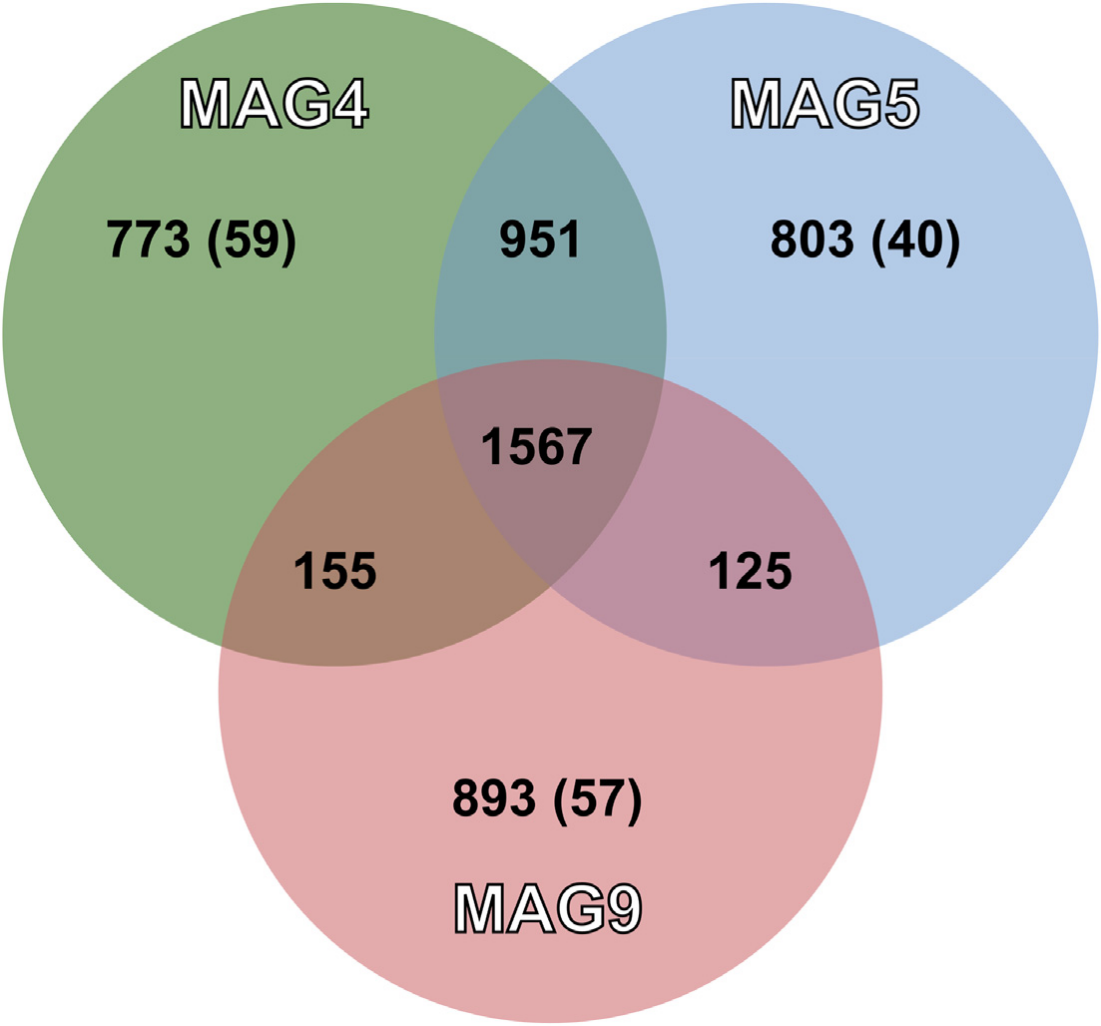
OrthoVenn2 diagram displaying orthologous gene clusters shared between the methanogen MAGs. The unique numbers in each circle display the singletons and between brackets paralogous gene clusters.

Overall, we observed clear distinctions between the three annotated *Methanosarcinaceae* MAGs. To investigate MAG-specific differences, we performed genome-wide nucleotide, amino acid and gene-based analyses (Fig. 3; see Table S4, Supporting Information). Due to the close identity of all *Methanosarcinaceae* MAGs to the genome of the psychrotolerant methanogen *M. lacustris*, this genome was used as a reference for the average nucleotide identity (ANI) and AAI calculations (see Table S4, Supporting Information). The ANI identity between the MAGs indicates that the methanogen MAGs may belong to different genera. The functional gene analyses do, however, indicate closer taxonomic affiliation. It has to be noted that the results on the absence of genes have to be interpreted with caution due to the fragmented nature and incompleteness of the MAGs.

Orthologous gene cluster analysis highlighted a large shared fraction between the three *Methanosarcinaceae* MAGs. A large additional number of gene clusters is shared between MAG 4 and MAG 5. These data are supported by genome-wide ANI and AAI values (see Table S4, Supporting Information). MAG 4 and MAG 5 are more similar (AAI: 87.8%) and MAG 9 shows higher dissimilarity (AAI: 78.0% to MAG 4 and 78.1% to MAG 5, respectively). Comparison with *M. lacustris* indicated closest identity of MAG 4 and MAG 5 to *M. lacustris* (AAI of 88.6% and 86.0%, respectively), whereas MAG 9 was more dissimilar (AAI of 77.4%).

### Increased acetate and TMA availability can boost methane production from thermokarst lake sediments

Overall, we observed a strong enrichment in methanogenic archaea upon acetate and TMA amendment of thermokarst lake sediments. An acetate conversion efficiency of 50% to CH_4_ together with an increase in acetoclastic *Methanosaetaceae/Methanotrichaceae* indicates the establishment of a stable methanogenic community (de Jong *et al*. 2018). Our initial 16S rRNA gene-based study was, however, not detailed enough to unravel potential species-specific responses. Upon metagenomic sequencing, we found that several bacterial species could support the methanogenic community by potentially increasing acetate availability. These results highlight that an increase in acetate availability could likely result in elevated CH_4_ production in the long term. This is highly relevant in the context of a warming Arctic (Mack *et al*. 2004; Herndon *et al*. 2015).

The strong increase of *Methanosarcinaceae* on TMA, together with an efficient substrate conversion efficiency of 73%, highlights their success upon an increased availability of methylated compounds in thermokarst lake sediments. Interestingly, the different responses of the MAGs to the temperature scenarios stressed that unique methanogenic species are responsible for methanogenesis at different temperatures. This change could only be unraveled with our metagenomics sequencing approach.

Methanogens are part of a complex microbial community. Due to intricate species interactions, the methanogenesis potential is partially controlled by the activity of fermentative microorganisms. In turn, the fermentative processes are controlled by several ecosystem characteristics, including pH, temperature and organic matter types (Bastviken *et al*. 2004; Ye *et al*. 2012; Roy Chowdhury *et al*. 2015). Here, we provided a link between the complex bacterial community and methanogenic archaea in thermokarst lake sediments using a controlled setup. Future research on the link between temperature, substrate availability and species dynamics *in situ* is highly needed, since this ultimately determines the ecosystem CH_4_ fluxes. Understanding these dynamics is important to better comprehend the mechanisms that underlie GHG production from Arctic lakes in a warming world.

## ACKNOWLEDGEMENTS

We thank Anniek de Jong, Ove Meisel, Joshua Dean and Han Dolman for the fruitful collaboration, inspiring discussions and novel discoveries.

## DATA AVAILABILITY

All sequencing data underlying this article are available in the GenBank databases at https://www.ncbi.nlm.nih.gov/bioproject/?term=prjna436632 an can be accessed under BioProject PRJNA436632 and at BioSample SAMN14764579–SAMN14764590.

## Supplementary Material

xtaa008_Supplemental_File

xtaa008_Reviewer_Comments_and_Author_Response
