# Supplementary material for: Long-term enriched methanogenic communities from thermokarst lake sediments show species-specific responses to warming
Source: FEMS Microbes. 2020 Oct 24;1(1):xtaa008. doi: 10.1093/femsmc/xtaa008 (PMC10117432; doi:10.1093/femsmc/xtaa008)
Supplement: xtaa008_Supplemental_File [file xtaa008_supplemental_file.docx]

**Supporting Information**

***Supplementary Table 1.*** *Fractional abundance of the archaeal 16S rRNA gene data from metagenomic datasets of the methanogenic incubations with acetate, trimethylamine (TMA) and control incubations at 4°C and 10°C. The group “Others” includes all taxonomic groups with a relative abundance <1%. Taxonomic identification is given up to Family level. 100% represents the total of 16S rRNA gene reads that were identified as archaeal reads.*

| Incubation  Taxonomy | **4°C Acetate** | **10°C Acetate** | **4°C  TMA** | **10°C TMA** | **4°C Control** | **10°C Control** |
| --- | --- | --- | --- | --- | --- | --- |
| *Methanosaetaceae* | 0.48 | 0.71 | 0.02 | 0.10 | 0.42 | 0.52 |
| *Methanosarcinaceae* | 0.28 | 0.14 | 0.96 | 0.77 | 0.07 | 0.05 |
| *Methanoperedenaceae* | 0.01 | 0.00 | 0.00 | 0.00 | 0.00 | 0.03 |
| Rice Cluster II | 0.05 | 0.05 | 0.00 | 0.03 | 0.10 | 0.07 |
| *Methanoregulaceae* | 0.07 | 0.03 | 0.01 | 0.05 | 0.04 | 0.17 |
| *Methanomassiliicoccaceae* | 0.01 | 0.02 | 0.00 | 0.03 | 0.03 | 0.04 |
| *Methanobacteriaceae* | 0.00 | 0.00 | 0.00 | 0.00 | 0.01 | 0.00 |
| Bathyarchaeia | 0.04 | 0.02 | 0.00 | 0.02 | 0.13 | 0.11 |
| Woesearchaeia | 0.04 | 0.01 | 0.00 | 0.00 | 0.16 | 0.02 |
| Micrarchaeia | 0.00 | 0.01 | 0.00 | 0.00 | 0.03 | 0.00 |
| Others | 0.02 | 0.03 | 0.02 | 0.00 | 0.03 | 0.00 |
| % of total reads | 0.11 | 0.13 | 0.50 | 0.33 | 0.04 | 0.04 |

***Supplementary Table 2.*** *Frequency data of the bacterial 16S rRNA gene data from metagenomic datasets of the methanogenic incubations with acetate, trimethylamine (TMA) and control incubations at 4°C and 10°C. The group “Others” includes all taxonomic groups with a relative abundance <1%. Taxonomic identification is given up to Order level.*

| Incubation  Taxonomy | **4°C Acetate** | **10°C Acetate** | **4°C  TMA** | **10°C TMA** | **4°C Control** | **10°C Control** |
| --- | --- | --- | --- | --- | --- | --- |
| Desulfuromonadales | 0.14 | 0.10 | 0.07 | 0.07 | 0.06 | 0.05 |
| Syntrophobacterales | 0.04 | 0.04 | 0.03 | 0.04 | 0.04 | 0.05 |
| Myxococcales | 0.02 | 0.01 | 0.02 | 0.01 | 0.02 | 0.02 |
| Desulfobacterales | 0.01 | 0.02 | 0.03 | 0.02 | 0.05 | 0.05 |
| Betaproteobacteriales | 0.07 | 0.07 | 0.05 | 0.07 | 0.08 | 0.06 |
| Methylococcales | 0.01 | 0.01 | 0.01 | 0.01 | 0.01 | 0.01 |
| Clostridiales | 0.09 | 0.17 | 0.23 | 0.17 | 0.11 | 0.08 |
| Selenomonadales | 0.00 | 0.00 | 0.08 | 0.00 | 0.03 | 0.01 |
| Anaerolineales | 0.09 | 0.08 | 0.08 | 0.09 | 0.08 | 0.11 |
| SJA-15 | 0.00 | 0.00 | 0.00 | 0.00 | 0.00 | 0.01 |
| KD4-96 | 0.02 | 0.03 | 0.01 | 0.02 | 0.02 | 0.05 |
| Dehalococcoidia | 0.01 | 0.01 | 0.01 | 0.00 | 0.01 | 0.01 |
| Bacteroidales | 0.20 | 0.19 | 0.19 | 0.20 | 0.16 | 0.14 |
| Sphingobacteriales | 0.01 | 0.01 | 0.01 | 0.01 | 0.01 | 0.01 |
| Gaiellales | 0.01 | 0.00 | 0.00 | 0.00 | 0.01 | 0.02 |
| Aminicenantales | 0.01 | 0.01 | 0.01 | 0.02 | 0.01 | 0.03 |
| Solibacteriales | 0.00 | 0.00 | 0.01 | 0.00 | 0.01 | 0.01 |
| Subgroup 6 | 0.00 | 0.00 | 0.00 | 0.01 | 0.01 | 0.01 |
| Microgenomatia | 0.01 | 0.01 | 0.01 | 0.01 | 0.02 | 0.01 |
| Patescibacteria | 0.00 | 0.00 | 0.00 | 0.02 | 0.00 | 0.00 |
| Parcubacteria | 0.01 | 0.00 | 0.00 | 0.00 | 0.00 | 0.00 |
| Phycisphaerales | 0.01 | 0.01 | 0.01 | 0.02 | 0.00 | 0.00 |
| Pedosphaerales | 0.01 | 0.01 | 0.00 | 0.01 | 0.01 | 0.01 |
| Spirochaetales | 0.01 | 0.00 | 0.02 | 0.01 | 0.00 | 0.01 |
| Others | 0.25 | 0.23 | 0.18 | 0.21 | 0.29 | 0.29 |
| % of total reads | 0.86 | 0.84 | 0.48 | 0.65 | 0.91 | 0.93 |

***Supplementary Table 3.*** *Characteristics of the 10 metagenome-assembled genomes (MAGs) with an estimated genome completeness >70%. Taxonomy shows the taxonomy of the MAG as assessed by the GTDB-Tk toolkit. Completeness, redundancy, strain heterogeneity, GC content and genome size were assessed by CheckM.*

| # | Taxonomy | Completeness | Redundancy | Strain heterogeneity | GC content | Genome size (Mbp) | Number of contigs | n50 Contigs |
| --- | --- | --- | --- | --- | --- | --- | --- | --- |
| 1 | Pelobacteraceae (Family) | 100.0% | 0.7% | 0.0% | 52.4% | 3.67 | 122 | 58,623 |
| 2 | Bacteroidales (Order) | 99.5% | 3.8% | 9.1% | 40.6% | 4.94 | 116 | 103,013 |
| 3 | Peptostreptococcales (Order) | 99.3% | 2.5% | 11.1% | 49.0% | 3.79 | 121 | 72,016 |
| 4 | Methanosarcinaceae (Family) | 97.2% | 1.0% | 0.0% | 42.7% | 4.37 | 274 | 29,718 |
| 5 | Methanosarcinaceae (Family) | 96.8% | 0.0% | 0.0% | 42.0% | 4.27 | 210 | 34,121 |
| 6 | Bacteroidales vadinHA17 (Order) | 96.6% | 8.5% | 40.0% | 41.3% | 4.69 | 890 | 7,335 |
| 7 | Elusimicrobiales (Order) | 88.8% | 5.1% | 40.0% | 60.1% | 2.95 | 253 | 18,227 |
| 8 | Elusimicrobiales (Order) | 86.0% | 2.3% | 66.7% | 60.9% | 3.63 | 662 | 6,803 |
| 9 | Methanosarcinaceae (Family) | 84.5% | 2.0% | 50.0% | 40.8% | 3.54 | 917 | 4,407 |
| 10 | Anaerolineales (Order) | 77.6% | 4.0% | 42.9% | 54.8% | 2.82 | 783 | 4,091 |

***Supplementary Table 4****. Average nucleotide identity (ANI) and average amino acid identity (AAI), calculated with ANI/AAI-Matrix tool of Kostas Lab using default parameters, on 20-05-2019.*

| **MAGs** | **ANI %** | **AAI %** |
| --- | --- | --- |
| MAG 4 and MAG 5 | 89.4% | 87.8% |
| MAG 4 and MAG 9 | 81.3% | 78.0% |
| MAG 5 and MAG 9 | 81.3% | 78.1% |
| MAG 4 and *M. lacustris* | 91.4% | 88.6% |
| MAG 5 and *M. lacustris* | 88.3% | 86.0% |
| MAG 9 and *M. lacustris* | 80.8% | 77.4% |

***Supplementary table 5.*** *BLASTP identification of heterodisulfide reductase subunit D and E (HdrDE) sequences in Methanosarcinaceaea MAGs 4, 5 and 9.*

| **MAG** | **Annotated gene (Prokka)** | **BLASTP hit to Methanosarcina sp. HdrDE reference sequences** | **E-value** | **% aa identity** |
| --- | --- | --- | --- | --- |
| MAG 4 | EJGLFDCG_03142 MULTISPECIES: (Fe-S)-binding protein [*Methanosarcina*] | **HdrD** Q8TSV7 Dihydromethanophenazine:CoB--CoM heterodisulfide reductase subunit D OS=*Methanosarcina acetivorans* (strain ATCC 35395 / DSM 2834 / JCM 12185 / C2A | 0.00 | 94.9% |
| MAG 4 | EJGLFDCG_03143 hypothetical protein [*Methanosarcina* sp. 2.H.A.1B.4] | **HdrE**  Q8TSV8  Dihydromethanophenazine:CoB--CoM heterodisulfide reductase subunit E OS=*Methanosarcina acetivorans* (strain ATCC 35395 / DSM 2834 / JCM 12185 / C2A | 0.00 | 89.6% |
| MAG 5 | ECNOOHKJ_02014 disulfide reductase [*Methanosarcina lacustris*] | **HdrE**  Q8TSV8  Dihydromethanophenazine:CoB--CoM heterodisulfide reductase subunit E OS=*Methanosarcina acetivorans* (strain ATCC 35395 / DSM 2834 / JCM 12185 / C2A | 1.19e-179 | 90.4% |
| MAG 5 | ECNOOHKJ_02015 MULTISPECIES: (Fe-S)-binding protein [*Methanosarcina*] | **HdrD** Q8TSV7 Dihydromethanophenazine:CoB--CoM heterodisulfide reductase subunit D OS=*Methanosarcina acetivorans* (strain ATCC 35395 / DSM 2834 / JCM 12185 / C2A | 0.00 | 94.9% |
| MAG 9 | FCAKDNNF_00688 MULTISPECIES: disulfide reductase [*Methanosarcina*] | **HdrE**  Q8PVW4  Dihydromethanophenazine:CoB--CoM heterodisulfide reductase subunit E OS=*Methanosarcina mazei* (strain ATCC BAA-159 / DSM 3647 / Goe1 / Go1 / JCM 11833 / OCM 88 | 1.69e-166 | 83.9% |

***Supplementary Table 6.*** *Gene scoring for key genes in carbon fixation pathways. Green color indicates the complete gene cluster was detected* (occurrence frequency ≥1)*, yellow color indicates an incomplete gene cluster was detected. coo: carbon monoxide dehydrogenase, cdh: acetyl-CoA synthase, kor: 2-oxoglutarate oxidoreductase, acl: ATP citrate lyase, por: pyruvate:ferredoxin oxidoreductase, rbc: ribulose bisphosphate carboxylase/oxygenase. The numbers in the yellow cells indicate the completeness of the cluster.*

|  | **Wood-Ljungdahl** | | **Reverse TCA** | | | **CBB** |
| --- | --- | --- | --- | --- | --- | --- |
| MAG | *cooFS* | *cdhABCD* | *korABCD* | *aclAB* | *porABDG* | *rbcSL* |
| *Pelobacteraceae* (Family) | 0.5 |  |  |  | 0.25 |  |
| Bacteroidales (Order) |  |  |  |  |  |  |
| Peptostreptococcales (Order) |  |  |  |  |  |  |
| *Methanosarcinaceae* (Family) |  |  | 0.75 |  |  | 0.5 |
| *Methanosarcinaceae* (Family) |  | 0.75 | 0.75 |  |  | 0.5 |
| Bacteroidales vadinHA17 (Order) | 0.5 |  |  |  |  |  |
| Elusimicrobiales (Order) |  |  |  |  |  |  |
| Elusimicrobiales (Order) |  |  |  |  | 0.25 |  |
| *Methanosarcinaceae* (Family) | 0.5 | 0.75 |  |  |  |  |
| Anaerolineales (Order) |  |  |  | 0.5 | 0.25 |  |

***Supplementary Table 7.*** *Gene scoring for key genes in fermentation pathways. Green color indicates the complete gene cluster was detected* (occurrence frequency ≥1)*, yellow color indicates an incomplete gene cluster was detected. hxl: 3-hexulose-6-phosphate synthase, hps-phi: bifunctional 3-hexulose-6-phosphate formaldehyde lyase/6-phospho-3-hexuloisomerase, fae-hps: bifunctional formaldehyde-activating enzyme/3- hexulose-6-phosphate synthase, gly: serine hydroxymethyltransferase, ack: acetate kinase, buk: butyrate kinase, pfl: pyruvate formate-lyase, ldh: lactate dehydrogenase. The numbers in the yellow cells indicate the completeness of the cluster.*

|  | **Ribulose monophosphate pathway** | | **Serine pathway** | **Acetate** | **Butyrate** | **Pyruvate/Formate** | |
| --- | --- | --- | --- | --- | --- | --- | --- |
| MAG | *hxlAB* | *hps-phi & fae-hps* | *glyA* | *ackA* | *buk* | *pflD* | *ldhA / ldh* |
| *Pelobacteraceae* (Family) |  |  |  |  |  |  |  |
| Bacteroidales (Order) |  |  |  |  |  |  |  |
| Peptostreptococcales (Order) |  |  |  |  |  |  |  |
| *Methanosarcinaceae* (Family) | 0.5 |  |  |  |  |  |  |
| *Methanosarcinaceae* (Family) | 0.5 |  |  |  |  |  |  |
| Bacteroidales vadinHA17 (Order) |  |  |  |  |  |  |  |
| Elusimicrobiales (Order) |  |  |  |  |  |  |  |
| Elusimicrobiales (Order) |  |  |  |  |  |  |  |
| *Methanosarcinaceae* (Family) | 0.5 | 0.5 |  |  |  |  |  |
| Anaerolineales (Order) |  |  |  |  |  |  |  |

|  | **Nitrogen fixation** | | **Dissimilatory**  **sulfate reduction** | | **Dissimilatory**  **sulfite**  **reduction** | **Sulfur oxidation** | **Sulfite oxidation** | |
| --- | --- | --- | --- | --- | --- | --- | --- | --- |
| MAG | *nifDHK* | *nrfA* | *cysND* | *aprAB* | *dsrAB* | *soxABXYZ* | *SorAB* | *yedY* |
| *Pelobacteraceae* (Family) |  |  |  |  |  |  |  |  |
| Bacteroidales (Order) |  |  | 0.5 | 0.5 |  | 0.2 |  |  |
| Peptostreptococcales (Order) |  |  |  |  |  |  |  |  |
| *Methanosarcinaceae* (Family) |  |  | 0.5 | 0.5 |  |  |  |  |
| *Methanosarcinaceae* (Family) | 0.67 |  | 0.5 |  |  |  |  |  |
| Bacteroidales vadinHA17 (Order) |  |  |  |  |  |  |  |  |
| Elusimicrobiales (Order) |  |  | 0.5 |  |  | 0.2 |  |  |
| Elusimicrobiales (Order) |  |  |  |  |  | 0.2 |  |  |
| *Methanosarcinaceae* (Family) |  |  | 0.5 | 0.5 |  |  |  |  |
| Anaerolineales (Order) |  |  |  |  |  |  |  |  |

***Supplementary Table 8.*** *Gene scoring for key genes in the nitrogen and sulfur cycle. Green color indicates the complete gene cluster was detected* (occurrence frequency ≥1)*, yellow color indicates an incomplete gene cluster was detected. nif: nitrogenase, nrf: cytochrome c nitrite reductase, cys: sulfate adenylyltransferase, apr: adenylylsulfate reductase, dsr: sulfite reductase, sox: sulfur oxidation pathway, sor: sulfite oxidase, yed: sulfite oxidase. The numbers in the yellow cells indicate the completeness of the cluster.*
