# Supplementary material for: Long-term enriched methanogenic communities from thermokarst lake sediments show species-specific responses to warming
Source: FEMS Microbes. 2020 Oct 24;1(1):xtaa008. doi: 10.1093/femsmc/xtaa008 (PMC10117432; doi:10.1093/femsmc/xtaa008)
Supplement: xtaa008_Reviewer_Comments_and_Author_Response [file xtaa008_reviewer_comments_and_author_response.docx]

Reviewer 1: Anonymous

Comments to the Author

This is a well-written and presented paper. The methods/ analysis approach used seem appropriate to the stated objective. The paper reports on a metagenomics analysis of DNA extracted from methanogenic enrichments supplied with acetate, TMA or no methanogenic substrate and incubated at 4 or 10oC. These experiments and the methane produced from them have been described previously and this earlier paper had carried out a prokaryote community analysis based on 16S rRNA gene sequence analysis. This follow on metagenomics study is justified by the statement on line 83 that ‘This [previous] dataset can, however, not provide in-depth insights into changes in both species composition and metabolic potential of the microbial communities exposed to the combined warming and substrate amendment scenario’. I have two major issues with this statement and the subsequent results and discussion.
1. Firstly, What is the justification for this statement –for instance, the obvious question to resolve is with regards to the Methanosarcinacae. The authors have not demonstrated whether the Methanosarcinacea (which are found in the different enrichments) have (within the region amplified and sequenced) identical 16S rRNA sequences. On this basis, therefore, the community cannot be discriminated by a straightforward community analysis - Are the authors explicitly saying that all the Methanosarcinacea sequences indicated in figure 1 of this paper (or in community analysis in the previous de Jong paper) all have 100% sequence homology with each other across all the experiments. If not are their patterns of different enrichment for different OTUs (ASVs) assignable to different families.
2. Secondly, even if the statement about the inadequacy of the community analysis approach can be properly justified. What are the actual new insights gained regarding the future response of methanogenic communities to climate warming that are not inferable simply from the taxonomic assignments made based on 16S rRNA (see specific comments on different sections of the discussion where this point is made more specifically).
Specific comments
3. Line 41 was this checked by running 16S rRNA based analysis on these same samples where individual OTUs within a family or genus were compared across different putative climate induced changes in enrichment conditions.
4. Line 51 ‘34’ what are warming potential units??? GWP??? - This seems higher than other values I have seen in the literature- why have the authors chosen this value
5. Line 88 there are plenty of examples of observing shifts in bacterial and archaeal taxa and inferring their function based simply on community analysis
6. Line 97is this an unnamed lake or is it named ‘unnamed lake’
7. Line 107 mM by convention implies a concentration but this is meaningless with respect how much was added unless you also indicate the total volume. Why not provide the amounts in mMoles. In terms of conversion of these additions to methane was a yield relative to the amount added calculated based on the stoichiometry of acetoclastic and methylotrophic methanogenesis –is this what conversion efficiency means when used later in the paper
8. Line 110 can the authors be more specific- a single DNA extract comprised of a slurry mixture of the three replicate of an experimental enrichment resulting in 6 extracts and 6 metagenome libraries- this becomes clear later in the paper of course but for the readers ease?
9. Line 119 approx. Why the two extraction methods. Did I miss somewhere where these different extracts are combined? Its not clear how these duplicates are used in the prep of the 6 metagenome libraries
10. Line 150 ???? why are these parenthesis brackets included
11. Line 156 Proteins ‘of’ ???? special interest for…..
12. Line 180 The authors should give a summary of the depth of the sequencing effort - the number of reads obtained for each library and an explicit statement that six sequence libraries were produced (see above)
13. Line 184 onwards – is the acetoclastic or catabolically versatile nature of these methanogens not require a reference to support the assumption
14. Line 185 How significant are these changes – I realise there is no replication of the metagenomes here but is this 1.7 supported by increased consumption of the added substrate as reflected by higher methane yields in the replicated experiments
15. Line 193 –what about some reference to the community composition as assessed by 16S rRNA in the last paper
16. Line 194 ‘decrease’ this is very confusing because I do not see a decrease in relation to acetate amendment. At least relative to the controls I see an increase -have I misunderstood -OK I see that you are saying decrease with temperature but it is odd when the sentence began ‘On acetate….’ that you flag this temperature change and ignore the larger difference of with or without acetate. This does not get said until line 223
17. Line 200 I think discussion of the shifts in bacterial populations are meaningless as they are relatively minor in scale and you have not done any replication
18. Line 216 the anaerobic character of the bacteria can be inferred based on the 16S rRNA sequences alone and presumably this was possible in the original community analysis – given there is little evidence of any enrichment presumably these same bacteria were observed in the original core material
19. Line 224 Was a metagenome necessary to make the statement ‘It has been shown for several Pelobacter species that acetate is required as carbon source for growth, but that it is not used in central energy metabolism (Schink 1985; Lovley et al. 1995; Richter et al. 2007).
20. Line 227 but given that you can identify this change in pelobacteracae from the 16S rRNA sequences and you could, based on this change, make the same reference to the known cultured peloba... what was the bennefit of closing out the genome - how many organoheterotrophic bacteria do not possess acetate symporters or have an acetate kinase If it is assumed that these bugs are growing on acetate what do the authors think is the mechanism - what is the oxidant or reductant since acetate cannot be fermented. It could be oxidized to CO2 if hydrogen is produced (syntrophic acetate oxidation - can it be determined that a large proportion of the acetate was not converted to methane - what do the mass balance equations say about how much acetate might be available if it is not converted to hydrogen and then converted to methane.
21. Line 229 OK but again this insight i.e. lack of temperature response does not come from doing metagenomics community analysis will do the trick.
22. Line 239 an obvious question not asked here is- do any of the Pelobacter or more generally desulfuromondales genomes known not contain cytochrome c3. Is the presence of this gene something unexpected or unusual for what is known for these organims –indeed how common is a cytochrome C3 more broadly –so is the insight that this organism is oxidizing acetate by metal reduction in these incubations –a cryptic comment about metal depletion is made but not explicitly elaborated on here. I think what the authors are trying to say is that Pelobacter are not likely to have actually been reducing metals in the microcosm because no exogenous electron acceptor was added
23. Line 241 why is elemental sulfur thrown into this sentence without explanation - I assume that the authors are implying sulfur as an electron acceptor here -this gets no support or explanation in the manuscript.
24. The overall point here is that it is widely recognised that Pelobacter are capable of iron and sulphur reduction so this could be inferred from community analysis and it is hardly a shock to find a cytochrome c3. I do not want to be a kill joy here and the observation of Cytochrome C3 is certainly interesting but this is surely not the insight that required a metagenome approach
25. Line 249 this section on the co-occurrence of pelobacter with methanogens is extremely vague. I assume that the authors are trying to imply the role of pelobacter in syntrophic acetate oxidation to hydrogen and CO2 - but if this was the case can they indicate the hydrogenotrophic methanogen that might be enriched to support such a process. I suppose this hydrogenotrophic function can be ascribed to the enriched Methanosarcinaceae but the authors do not discuss this here
26. Line 256 but none of this required the metagenome to provide the insight Line 260 - which is why it would have been useful to comment on what was observed in original community before incubation
27. how many genomes of bacterial anaerobes do not possess such genes – e.g. acetyl-CoA synthase. Acetate kinase is important in glycolysis –what is the great insight here
28. line 278 again no insight required from the metagenome
29. line 281 were these Elusimicrobiales sequences not observed based on their 16S rRNA sequences -they do not appear in table S2 -but presumably these are in the libraries and can be found in the original community analysis
30. line 284 why was it interesting that you identified nitrite reductase in these methanogenic enrichments. This organism clearly was not participating in the pathway leading to the generation of methane in a warming climate which is stated subject of this paper
31. line 289 We could also reconstruct an Anaerolineales…. Change the tense of this sentence.
32. Line 305 The stated purpose of this paper was ‘application of a full metagenome sequencing on long-term thermokarst lake sediment enrichments on acetate and trimethylamine at 4°C and 10°C to unravel species-specific responses to the most likely Arctic climate change scenario and specifically those involved in methanogenesis’. The previous section on Anaerolineales and Elusimicrobia does not address this point because these are not affected by the incubation. Also It should be remembered that relative abundance is a zero sum game so the increase in methanogens from growth on addition of acetate or TMA will by default decrease the relative abundance of other species unless they themselves are able to grow.
33. Line 316 onwards this is all inferable from 16S rRNA
34. Line 325-329 all this did not require a genome insight
35. Line 344 Nitrogen fixation is well known in many methanogenic archaea. The ability to fix N2 exists in Methanosarcina acetovorans, Methanosarcina etc ....what complete genomes has it not been observed
36. Line 351 so are the 16S rRNA gene sequences you have for each of the MAGS identical supporting the need to discriminate based on this metagenomic approach - -how many distinct ASVs were identified in the original community analysis. Maybe you do not have 16S rRNA for MAGS9 but presumably you do for 4 and 5.
37. Line 357 so what genes identifiable in the MAGS confirm that these organisms have adaptations to low temperature
38. Line 372 -373 is this surprising given that this is a Methanosarcinaceae - are there genomes of Methanosarcinaceae which do not have these genes
39. Line 374 so in the original 2018 study was there not a methanosarcina 16SrRNA sequence type which was specifically enriched in the TMA amended experiments in relation to some methanosarcina OTUs that were not
40. Line 392 again is this surprising given that this is a Methanosarcinaceae - are there genomes of Methanosarcinaceae which do not have these genes
41. Line 400 is absence of evidence of absence for MAG9 -this genome is only 85% complete
42. Line 403 the way I read this sentence is that Welte and Deppenmeier 2014 could not detect the sodium ion translocating Rnf complex in MAG9 but I do not think this is what the authors mean – additionally again is the absence real or a sampling effort problem
43. Line 409 antiviral defenc(s)e mechanism? what insight into the theme of the paper does this provide with respect to substrate or temperature
44. Line 413 approx. at this point the paper is simply describing some genes found and there is no obvious purpose to their mention
45. Line 425 It is well known that methanosarcina can participate in syntrophy with hydrogen producing organisms. But MAG 4 and MAG5 are enriched in the TMA amended microcosms so what are the authors trying to say here. Surely the enrichment is based on direct consumption of TMA to produce methane -this is quite confusing
46. Line 454 was this was not possible from knowing the bacterial community structure - it was not clear what this support was - acetate is a substrate for acetoclastic methanogens so when you added it why would the methanogens need any support under increased acetate availability. It is pretty obvious that we have, just from a community analysis, the presence of obligate and facilitative anaerobes that are capable of the breakdown and fermentation of organic matter producing acetate and hydrogen to drive methanogenesis. Warming which releases this organic matter will therefore end up supplying these products of fermentation for methanogens
47. Line 459 but this is reporting on what would have been known previously in the 2018 paper
48. Table S1 Frequency is the number of occurrences of a repeating event per unit of time -do the authors mean fractional abundances of 16S rRNA gene sequences . in the table I think a number like 0.48 -is a fractional abundance -this is not said in the table anywhere and if so what is it a fractional abundance of? what total reads do you mean? total archaeal reads -this does not make sense

Reviewer 2: Anonymous

Comments to the Author

Summary
The manuscript titled “Long-term enriched methanogenic communities from thermokarst lake sediments show species-specific responses to warming” describes the differences in microbial community composition in enrichments from two thermokarst lake sediments (near Utqiagvik, Alaska). Metagenomic reads corresponding to the 16S rRNA gene were used to describe taxonomic composition and relative abundance of members of the microbial community. Additionally, genome reconstruction from metagenomes highlighted the presence of different members of the Methanosarcinaceae in TMA enrichments at two temperatures (4 and 10 ○C). This work confirms findings from a previous study that combined enrichments with substrates for methanogenesis and methanotrophy, 16S rRNA gene iTag sequencing, and qPCR to characterize the composition of the microbial community on the same two thermokarst lake sediments (de Jong et al. 2018). The authors now expand on that work providing insight into compositional differences in methanogenic enrichments as a response to the different temperatures, and describe the metabolic potential of metagenome-assembled genomes (MAGs) representative of abundant members of the community. The manuscript is well written and logical, the goals of the study were made clear, and the methods seem appropriate. However, discussion of ‘species-specific’ responses could be misleading, considering that the most current species definition based on ANI requires at least 95% similarity among genomes. The following comments are meant to improve the clarity of the manuscript in general.

Major Issues
1) To ensure reproducibility of the methods, clarification of metagenomics analyses is required.
• Line 140: to what extent methods described in in ’t Zandt et al. (2019) apply to this study?
o Do methods for 16S rRNA gene analysis apply here too? If so, were 16S reads assembled for this study? There is no mention of that step in the section describing 16S analyses (also see below).
o Line 159: Were MAGs annotated with Prokka using RefSeq and also submitted to KAAS for pathway annotation using the 40 most closely related organisms? What is considered a functional gene in the context of this study? If only genes in Table 2, please explain why these were chosen.
o Was read mapping done using the Burrow-Wheeler Aligner? What was the mismatch allowed?
• What is the reasoning behind annotating the genomes using a customs HMMs database and Prokka? Please provide brief explanation.
o Line 150: What proteins were included in the custom HMMs database? (a list in the methods or in the Supporting Information would be helpful). If it isn’t already, I suggest making this database publicly available. Also, note information missing in parentheses next to Prodigal in this line.
• Lines 142-149: Please clarify methods used to identify reads mapping to 16S rRNA genes.
o If 16S reads were identified using BLASTn (line 143) against SILVA Ref NR 99 132, what do you mean by “Mapping was done in the CLC Genomics Workbench” (lines 145-46)? If the CLC workbench software was used to identify 16S reads, please provide some detail regarding how this is done.
o Might be useful to add a few sentences to the discussion about how a BLAST search may have impacted the identification of 16S short reads (as opposed to a CMsearch-based approach more commonly used with metagenomics data that takes into account the 16S rRNA secondary structure).
o What exactly was SILVAngs used for (Line 147)? A sentence or two would suffice.

2) Average nucleotide identity among genomes that are highly similar (80-100%) to each other has been used to identify genomes at the sub-species level. Assuming that the species boundary falls within 95-96% ANI (https://msystems.asm.org/content/5/1/e00731-19 and references 8-11), genomes with ANI < 95% can be assumed to be related at a higher taxonomic level. Therefore, genomes with ANIs between ~ 81-91% (Supplementary Table 4) are unlikely representatives of “sub-species” and may not reflect “species-specific” responses. To my knowledge, these might represent genus-level relationships (https://doi.org/10.1038/s41587-020-0501-8). It is important to take this into account in the wording of statements about species-level responses.

3) It would be useful to provide a supplementary table with sequencing statistics, particularly the total number of reads obtained per sample and how many of those reads were determined to correspond to the 16S rRNA gene.

Minor Issues:
• Line 50: Units?
• Line 113: A brief explanation justifying the use of two different extraction methods would strengthen this section.
• Line 137: Based on lines 129-130, sequencing libraries were prepared for each one of the two DNA extractions per pooled triplicates. Were libraries from each extraction method pooled prior to sequencing? Or is this sentence referring to something else? Table 1 suggests that abundance was normalized by “averaging the total read corrected coverage data per DNA extraction method”, as if the extracts were sequenced separately.
• Line 215: Clarify what is a ‘high’ amount.
• Line 228: Supplementary Table 7?
• Line 230: One could argue that this depends on the definition of psychrophile used. It may be more appropriate to discuss alternative explanations, including the temperature dependence of enzymatic reactions (based on Q10 for example), enzymatic saturation at given substrate concentrations, or competition among community members.
• Line 271: how was the lack of potential for humic acids degradation established?
• Line 279: the authors may find this reference useful: https://doi.org/10.1038/s41396-020-0716-1.
• Line 360: Describe method used to identify MAGs species based on the mcrA gene. Was this based on phylogenetics?
• Line 376: Table 2 ->Table 1?
• Line 433: remove period after Methanosarcina.
• Throughout the text, it looks like some references were cited in support of findings from this study although no context was provided in most instances (e.g., line 272, line 402).
• Figures 1-2: What percent of the reads per sample correspond to the 16S rRNA gene? Would be useful to include this information on a separate table (see above) or in the figure legend.
• Table 1: Grouping genomes by taxon may facilitate comparisons among MAGs. How was the mapping used for abundance calculation done? (please list algorithms and parameters used in the methods).
• Table 2: Why was the comparison restricted to these genes? A couple of sentences would help to clarify.
• Supplementary Tables 1-2: Are these actual percentages or proportions? For instance, were 0.11% or 11% of the total number of 16S rRNA gene reads derived from the acetate-amended/4 °C metagenome identified as Archaea?
• Table 2 and Supplementary Tables 6-8: Please clarify definition of gene clusters. Do clusters correspond to genes encoding a whole enzyme, ‘key genes’ in a pathway, or all of the genes involved in a given pathway? Seems to vary across tables, it may be appropriate to address this in the methods.
o For instance, in Table 2: were all genes that make the Rnf complex included in the analysis? and if so, what proportion had to be present to indicate presence?
o Supplementary Table 7-Line 40: clustered -> cluster

Author’s Response:

**Author's Response to Decision Letter for (FEMSMC-2020-006)**

**Long-term enriched methanogenic communities from thermokarst lake sediments show species-specific responses to warming**

Dear editor,
We thank you for the expert handling of our manuscript and for the very detailed, valuable inputs and suggestions that benefited the manuscript. We have more explicitly mentioned the advantages of the metagenome approach compared to the 16S rRNA gene amplicon approach throughout the manuscript. Furthermore, we have better provided and highlighted the insights into the metagenomics datasets, with help of the comments of both reviewers. We once again wish to thank both reviewers for the detailed and very constructive feedbacks that helped to significantly improve our manuscript.
 
Reviewers' Comments to Authors:

Reviewer: 1

Comments to the Author
This is a well-written and presented paper. The methods/ analysis approach used seem appropriate to
the stated objective. The paper reports on a metagenomics analysis of DNA extracted from
methanogenic enrichments supplied with acetate, TMA or no methanogenic substrate and incubated at 4
or 10oC. These experiments and the methane produced from them have been described previously and
this earlier paper had carried out a prokaryote community analysis based on 16S rRNA gene sequence
analysis. This follow on metagenomics study is justified by the statement on line 83 that ‘This
[previous] dataset can, however, not provide in-depth insights into changes in both species
composition and metabolic potential of the microbial communities exposed to the combined warming
and substrate amendment scenario’. I have two major issues with this statement and the subsequent
results and discussion.

We thank reviewer #1 for the constructive, detailed inputs and suggestions on the manuscript. We have provided detailed responses to the comments below.

1. Firstly, What is the justification for this statement –for instance, the obvious question to
resolve is with regards to the Methanosarcinacae. The authors have not demonstrated whether the
Methanosarcinacea (which are found in the different enrichments) have (within the region amplified
and sequenced) identical 16S rRNA sequences. On this basis, therefore, the community cannot be
discriminated by a straightforward community analysis - Are the authors explicitly saying that all
the Methanosarcinacea sequences indicated in figure 1 of this paper (or in community analysis in
the previous de Jong paper) all have 100% sequence homology with each other across all the
experiments. If not are their patterns of different enrichment for different OTUs (ASVs) assignable
to different families.
>When clustering on family level the different ASVs cluster within the same family of Methanosarcinaceae as indicated in the legend. As we have indicated in our manuscript, the resolution of short stretch 16S rRNA amplicon sequences hampers indentification of species and especially strain heterogeneity. Furthermore, here we have used short, unassembled reads that were classified as 16S sequence-containing reads. Using this method, the reconstruction of ASVs is not feasible. To refrain from speculations with this data, we have used it for global family/order-level identification and refrain from assessments of genus/species/strain diversity.
2. Secondly, even if the statement about the inadequacy of the community analysis approach can be
properly justified. What are the actual new insights gained regarding the future response of
methanogenic communities to climate warming that are not inferable simply from the taxonomic
assignments made based on 16S rRNA (see specific comments on different sections of the discussion
where this point is made more specifically).
>See also the comment above on the limitations of a 16S rRNA gene-only approach. In addition, we have better highlighted the advantages of the full metagenomics approach (functional assessment, more quantitative data, better taxonomic resolution) in the manuscript, see also the responses to the specific comments below.


Specific comments
3. Line 41 was this checked by running 16S rRNA based analysis on these same samples where
individual OTUs within a family or genus were compared across different putative climate induced
changes in enrichment conditions.
>Indeed OTU differences can be used to highlight differences between temperatures and substrate amendments. However, what we want to highlight here is that these differences do not provide insights into potentially functional differences of different Methanosarcinaceae. To better highlight this we have rephrased line 41 to read: “These highlighted distinct and potentially functional climate-induced shifts could not be revealed with 16S rRNA gene-based analyses”.
4. Line 51 ‘34’ what are warming potential units??? GWP??? - This seems higher than other values I
have seen in the literature- why have the authors chosen this value
>This is the GWP value as reported by the most recent IPCC report of 2013. This value includes atmospheric feedback loops (e.g. the associated increase in water vapor and CO2 concentrations due to increased CH4 emissions).
The GWP provides the climate impact of a certain gas compared to the same amount of CO2. To clarify this here we have rewritten line 51-52 (now line 53-54) to read: “34 for 100 years (compared to the climate impact of the same quantity of CO2”
5. Line 88 there are plenty of examples of observing shifts in bacterial and archaeal taxa and
inferring their function based simply on community analysis
>Indeed, such assessments are often performed. However, short stretch 16S rRNA gene sequences are not suited for species composition and metabolic potential of the microbial communities as is stressed in, amongst other, this sentence. We therefore advocate to refrain from speculative 16S rRNA gene-based functional assessments.
6. Line 97is this an unnamed lake or is it named ‘unnamed lake’
>The name of this lake is “Unnamed Lake”. To clarify this we’ve rewritten both lake names to read (“Lake Emaiksoun” and “Unnamed Lake”.)
7. Line 107 mM by convention implies a concentration but this is meaningless with respect how much
was added unless you also indicate the total volume. Why not provide the amounts in mMoles. In
terms of conversion of these additions to methane was a yield relative to the amount added
calculated based on the stoichiometry of acetoclastic and methylotrophic methanogenesis –is this
what conversion efficiency means when used later in the paper
>We use mM concentrations since this is relevant in respect to the ecosystem and for comparability with other ecosystem- and laboratory-based studies. Indeed, we agree that for interpretation it is useful to include the sample volume (50 mL), we’ve therefore included this in the materials & methods in line 108-109: “All sediment slurries had a volume of 50 mL.”
8. Line 110 can the authors be more specific- a single DNA extract comprised of a slurry mixture of
the three replicate of an experimental enrichment resulting in 6 extracts and 6 metagenome
libraries- this becomes clear later in the paper of course but for the readers ease?
>We have clarified this here by adding the sentence “Each single DNA sample therefore represents a triplicate incubation.” After the sentence after line 110 (now line 116-117). And in the next sentence by changing “sample” to “pooled sample” to stress the DNA sample is pooled from each triplicate incubation.
9. Line 119 approx. Why the two extraction methods. Did I miss somewhere where these different
extracts are combined? Its not clear how these duplicates are used in the prep of the 6 metagenome
libraries
>The different extraction methods were used to increase the resolution and dimensions for the reconstruction of metagenome-assembled genomes. We agree that this is not clear in this part. Therefore we have added a sentence at line 133-134: “The two DNA extraction methods were used to improve the resolution for downstream metagenome-assembled genome (MAG) reconstruction.” And “In total 12 DNA samples were prepared for metagenome sequencing.” To highlight that in total 12 libraries were prepared.
10. Line 150 ???? why are these parenthesis brackets included
>These are commonly included for description of software tools. However, these are not needed here, we have deleted the parentheses.
11. Line 156 Proteins ‘of’ ???? special interest for…..
>We have changed it to “Proteins of special interest” (line 162 in current version)
12. Line 180 The authors should give a summary of the depth of the sequencing effort - the number
of reads obtained for each library and an explicit statement that six sequence libraries were
produced (see above)
>As indicated above and highlighted in the manuscript a total of 12 libraries were prepared (six unique samples x 2 unique extraction methods). We have provided a summary of sequencing depth per sample in line 184-186: “A total of 12 libraries were sequenced. The average library depth was 5.2 million reads with a sequence quality >Q30 for 73.5% of the reads.”
13. Line 184 onwards – is the acetoclastic or catabolically versatile nature of these methanogens not require a reference to support the assumption
>We have provided a reference for this statement: Conrad R. Control of microbial methane production in wetland rice fields. Nutr Cycl Agroecosystems 2002;64:59–69.
14. Line 185 How significant are these changes – I realise there is no replication of the
metagenomes here but is this 1.7 supported by increased consumption of the added substrate as
reflected by higher methane yields in the replicated experiments
>This is a very valid point, indeed due to technical limitations it cannot be statistically proven. We do see an initial increased response in methane production on acetate at 10 degrees Celsius, as indicated in our previous study (de Jong et al. paper in EM). However, this is not seen on the long term (for total methane production from the same quantity of provided substrate. With our current studies we cannot unravel these short and long-term responses, so we refrain from speculating on these differences.
15. Line 193 –what about some reference to the community composition as assessed by 16S rRNA in the
last paper
>We agree that it provides valuable insights to refer to the previous 16S rRNA gene-based study here. We have added the following sentence and referred to the data of the study by de Jong et al. (Figure S4):
“In our previous 16S rRNA-gene based study we also observed a dominance of Desulfuromonadales, Clostridiales and Bacteroidales in the incubations on acetate and TMA, Anaerolineales were abundant in all incubations, and Betaproteobacteriales were below the detection limit (<2% relative 16S rRNA gene read abundance) (Figure S4 of de Jong et al., 2018).” In line 205-209
16. Line 194 ‘decrease’ this is very confusing because I do not see a decrease in relation to
acetate amendment. At least relative to the controls I see an increase -have I misunderstood -OK I
see that you are saying decrease with temperature but it is odd when the sentence began ‘On
acetate….’ that you flag this temperature change and ignore the larger difference of with or
without acetate. This does not get said until line 223
>Indeed this can be confusing here. We have therefore rephrased it to read: “On acetate the clearest responses were a temperature-specific decrease of Desulfuromonadales” in line 209-210
17. Line 200 I think discussion of the shifts in bacterial populations are meaningless as they are
relatively minor in scale and you have not done any replication
>We agree that these observations are speculative at best. However, we want to highlight the most important observed differences in our study. To highlight this is not supported by statistical analyses (replicates) we have added the following sentence “However, these differences were only observed between single samples, and more in-depth analyses and replications are needed to statistically confirm our observations.” (Line 216-218)
18. Line 216 the anaerobic character of the bacteria can be inferred based on the 16S rRNA
sequences alone and presumably this was possible in the original community analysis – given there
is little evidence of any enrichment presumably these same bacteria were observed in the original
core material
>Indeed, the original communities already contained mainly anaerobic microorganisms, although also specific aerobic bacteria were observed (like aerobic methanotrophs), as is highlighted in the study by de Jong et al. Here, we, however, expose the sediments to prolonged strictly anoxic conditions which eliminates aerobic communities.
To highlight the observation of our previous study, we’ve added this to these sentences that now read:
“The anaerobic incubations both with and without substrate additions contained a high amount of anaerobic fermentative bacteria which were also dominant in the original cores (Garrity, Bell and Lilburn 2004) (Table 1, see Supplementary Table 7 and Figure 1 of de Jong et al. 2018).”
19. Line 224 Was a metagenome necessary to make the statement ‘It has been shown for several
Pelobacter species that acetate is required as carbon source for growth, but that it is not used in
central energy metabolism (Schink 1985; Lovley et al. 1995; Richter et al. 2007).
>If you want to confirm that the Pelobacter specie(s) observed in your study is able to metabolize acetate, you indeed need metagenomic sequencing data. 16S rRNA gene-based sequencing data does not provide information on the metabolic potential.
20. Line 227 but given that you can identify this change in pelobacteracae from the 16S rRNA
sequences and you could, based on this change, make the same reference to the known cultured
peloba... what was the bennefit of closing out the genome - how many organoheterotrophic bacteria
do not possess acetate symporters or have an acetate kinase If it is assumed that these bugs are
growing on acetate what do the authors think is the mechanism - what is the oxidant or reductant
since acetate cannot be fermented. It could be oxidized to CO2 if hydrogen is produced (syntrophic
acetate oxidation - can it be determined that a large proportion of the acetate was not converted
to methane - what do the mass balance equations say about how much acetate might be available if it
is not converted to hydrogen and then converted to methane.
>In the beginning of the incubations also minor amounts of alternative electron acceptors could play a role (e.g. nitrate and sulfate). Due to complex interactions and the fact that intermediate H2 could not be detected (rapid turnover) it is too speculative to assess and quantify potential conversion pathways of the acetate here. However, on the long-term syntrophic acetate oxidation is most likely, but we do not have experimental proof. We therefore refrain from speculating too much on this.
In addition, as highlighted above 16S rRNA gene-based analyses are not suitable for functional assessment of e.g. Pelobacter species.
21. Line 229 OK but again this insight i.e. lack of temperature response does not come from doing
metagenomics community analysis will do the trick.
>The potential to metabolize acetate as highlighted in the sentence above “The MAG reported here contains several genes encoding cation acetate symporters and an acetate kinase” can only be provided by metagenome analyses. The general increase is indeed not a unique feature of metagenomics data, but it does provide the general observation on changes with temperature.
22. Line 239 an obvious question not asked here is- do any of the Pelobacter or more generally
desulfuromondales genomes known not contain cytochrome c3. Is the presence of this gene something
unexpected or unusual for what is known for these organims –indeed how common is a cytochrome C3
more broadly –so is the insight that this organism is oxidizing acetate by metal reduction in these
incubations –a cryptic comment about metal depletion is made but not explicitly elaborated on here.
I think what the authors are trying to say is that Pelobacter are not likely to have actually been
reducing metals in the microcosm because no exogenous electron acceptor was added
>Indeed, we want to stress that metal reduction has probably not played a major role on the long term, due to expected depletion of alternative electron acceptors under the applied incubation conditions. To further highlight this we’ve added a sentence at line 259-260: “Since these alternative electron acceptors were not added, they probably played only minor roles.”
Regarding cytochrome c3, this specific and only observed cyt c3 was most identical to the one previously described by Lovley and co-workers (1995). It is beyond the scope of this study to further classify and compare these cytochromes.
23. Line 241 why is elemental sulfur thrown into this sentence without explanation - I assume that
the authors are implying sulfur as an electron acceptor here -this gets no support or explanation
in the manuscript.
>Elemental sulfur is mentioned due to the link of Desulfuromonadales with metal and elemental sulfur reduction as mentioned above: “Desulfuromonadales sequences have been linked to sulfur and
238 metal reduction”.
24. The overall point here is that it is widely recognised that Pelobacter are capable of iron and
sulphur reduction so this could be inferred from community analysis and it is hardly a shock to
find a cytochrome c3. I do not want to be a kill joy here and the observation of Cytochrome C3 is
certainly interesting but this is surely not the insight that required a metagenome approach
>The identification of a cytochrome c3 sequence cannot be done by using a 16S rRNA gene approach. A metagenome approach is needed for functional gene assessment. Indeed, the observation of e.g. a cytochrome c3 might not be unique, but it can be used for e.g. more specific taxonomic identification, comparison with previous studies (as done here) of species comparisons.
25. Line 249 this section on the co-occurrence of pelobacter with methanogens is extremely vague. I
assume that the authors are trying to imply the role of pelobacter in syntrophic acetate oxidation
to hydrogen and CO2 - but if this was the case can they indicate the hydrogenotrophic methanogen
that might be enriched to support such a process. I suppose this hydrogenotrophic function can be
ascribed to the enriched Methanosarcinaceae but the authors do not discuss this here
>Indeed, we specifically observed the link with Methanosarcinaceae. To indicate this and to better embed it with our study we’ve changed the sentence to read: “Here we also observed their co-occurrence with methanogenic archaea, more specifically with Methanosarcinaceae, in both substrate-amended and unamended sediments which highlights their potential methanogen-supporting role in organic-rich methanogenic ecosystems.” (line 266-269)
26. Line 256 but none of this required the metagenome to provide the insight Line 260 - which is
why it would have been useful to comment on what was observed in original community before
incubation
>Indeed, also 16S rRNA gene based studies can assess these differences, but this is one of the analyses that can be done with metagenomic datasets.
We have added a link to the original communities here, it reads: “The observation that Bacteroidales were more abundant in the original cores supports these observations (Figure 1 of de Jong et al. (2018)).” (line 281-282)
27. how many genomes of bacterial anaerobes do not possess such genes – e.g. acetyl-CoA synthase.
Acetate kinase is important in glycolysis –what is the great insight here
>Indeed acetate kinase is very common in anaerobes. Here we want to confirm that, as expected, indeed these metagenome-assembled genomes (MAGs) contain the genes for acetate metabolism. In addition, this confirms that the MAGs are of decent quality for functional assessment.
28. line 278 again no insight required from the metagenome
>Due to limited completeness of both Elusimicrobiales MAGs we could not perform in-depth functional assessment of these MAGs. The limited analyses that could be performed are presented in this paragraph.
29. line 281 were these Elusimicrobiales sequences not observed based on their 16S rRNA sequences
-they do not appear in table S2 -but presumably these are in the libraries and can be found in the
original community analysis
>Elusimicrobiales were below the detection limit in the 16S rRNA gene-based study on the original cores and the enriched samples (study of de Jong et al. (2018). Functional assessments are, however, not possible with these datasets. For this reason and the fact that little is known about the Elusimicrobiales, we performed the analyses that were possible, as described in this paragraph.
30. line 284 why was it interesting that you identified nitrite reductase in these methanogenic
enrichments. This organism clearly was not participating in the pathway leading to the generation
of methane in a warming climate which is stated subject of this paper
>Indeed, the observation of a nitrate reductase does not have a direct link with methane metabolism. However, there is a strong link between the carbon and nitrogen cycle under a changing climate. Here, due to sample limitations (limited completeness and resolution of the Elusimicrobiales MAGs) we performed the analyses that were possible. Their potential role in nitrogen cycling, in addition, does provide insights into their ecosystem function.
31. line 289 We could also reconstruct an Anaerolineales…. Change the tense of this sentence.
>Changed to “we also reconstructed”
32. Line 305 The stated purpose of this paper was ‘application of a full metagenome sequencing on
long-term thermokarst lake sediment enrichments on acetate and trimethylamine at 4°C and 10°C to
unravel species-specific responses to the most likely Arctic climate change scenario and
specifically those involved in methanogenesis’. The previous section on Anaerolineales and
Elusimicrobia does not address this point because these are not affected by the incubation. Also It
should be remembered that relative abundance is a zero sum game so the increase in methanogens from
growth on addition of acetate or TMA will by default decrease the relative abundance of other
species unless they themselves are able to grow.
>Indeed, both the Anaerolineales and Elusimicrobia are not directly linked to the methane cycle. However, three of the 10 most abundant MAGs were identified as Anaerolineales (1x) and Elusimicrobiales (2x), we therefore decided to shortly describe these MAGs and their functional potential that could be deduced from the available dataset.
33. Line 316 onwards this is all inferable from 16S rRNA
>Although 16S rRNA gene-based data can also be used, this is absolutely not the same. Where 16S rRNA gene-based amplicon sequencing is heavily biased by the selected primers and PCR amplification, random full-metagenome sequencing with several DNA extraction methods is much less biased. Therefore, metagenomic datasets provide much more accurate tools for abundance changes.
34. Line 325-329 all this did not require a genome insight
>Please see the above comment.
35. Line 344 Nitrogen fixation is well known in many methanogenic archaea. The ability to fix N2
exists in Methanosarcina acetovorans, Methanosarcina etc ....what complete genomes has it not been
observed
>Indeed, this is not a unique feature of our Methanosarcina MAG. However, we have used it here to confirm its potential and for species identification. It furthermore indicates the high quality and completeness of the MAG.
36. Line 351 so are the 16S rRNA gene sequences you have for each of the MAGS identical supporting
the need to discriminate based on this metagenomic approach - -how many distinct ASVs were
identified in the original community analysis. Maybe you do not have 16S rRNA for MAGS9 but
presumably you do for 4 and 5.
>Unfortunately, none of the methanogen MAGs contained a 16S rRNA gene, as is highlighted in line 382.
37. Line 357 so what genes identifiable in the MAGS confirm that these organisms have adaptations
to low temperature
>As highlighted in this sentence, their higher relative abundance at 10 degrees Celsius indicates their preference for higher temperatures, not of adaptation genes to low temperatures.
38. Line 372 -373 is this surprising given that this is a Methanosarcinaceae - are there genomes of
Methanosarcinaceae which do not have these genes
>These are common pathways for methanogenic archaea. Here, this data is provided as a general description of the methanogen MAGs and to highlight their potential as well as the completeness.
39. Line 374 so in the original 2018 study was there not a methanosarcina 16SrRNA sequence type
which was specifically enriched in the TMA amended experiments in relation to some methanosarcina
OTUs that were not
>The methanogen ASVs of the 2018 study were highly diverse and did not directly show a pattern as was observed by our study. In addition, our metagenome approach also had a much higher sequence resolution.
40. Line 392 again is this surprising given that this is a Methanosarcinaceae - are there genomes
of Methanosarcinaceae which do not have these genes
>As mentioned above, indeed these genes are general for Methanosarcinaceae. Here, we provided a general description of the MAGs, not necessarily highlighting novelty of this MAG.
41. Line 400 is absence of evidence of absence for MAG9 -this genome is only 85% complete
>Indeed, the genome is 85% complete, which implies absence can be caused by MAG incompleteness. We have highlighted this here: “The sodium ion translocating Rnf complex that is linked to energy conservation in Methanosarcinaceae could not be detected in MAG 9, probably due to genome incompleteness (Welte and Deppenmeier 2014).” (line 424)
42. Line 403 the way I read this sentence is that Welte and Deppenmeier 2014 could not detect the
sodium ion translocating Rnf complex in MAG9 but I do not think this is what the authors mean –
additionally again is the absence real or a sampling effort problem
>Indeed, this is confusing. We have therefore rephrased the sentence, see above.
43. Line 409 antiviral defenc(s)e mechanism? what insight into the theme of the paper does this
provide with respect to substrate or temperature
>There indeed is no direct link with substate or temperature effects. However, this was a universal observation of the different Methanosarcinaceae MAGs, and therefore in our opinion worthwhile to mention.
44. Line 413 approx. at this point the paper is simply describing some genes found and there is no
obvious purpose to their mention
>This part shortly describes the antiviral defense mechanisms that were detected in the different MAGs. Also see the above comment.
45. Line 425 It is well known that methanosarcina can participate in syntrophy with hydrogen
producing organisms. But MAG 4 and MAG5 are enriched in the TMA amended microcosms so what are the
authors trying to say here. Surely the enrichment is based on direct consumption of TMA to produce
methane -this is quite confusing
>Indeed, MAG4 and MAG5 are mainly enriched under TMA amendment, probably due to the non-competitive nature of TMA which is mentioned before. To better highlight this is mainly relevant under conditions where acetate is present, we have added the following: “This strategy can be relevant under environmental conditions in which acetate is increasingly available.” (line 450-451)
46. Line 454 was this was not possible from knowing the bacterial community structure - it was not
clear what this support was - acetate is a substrate for acetoclastic methanogens so when you added
it why would the methanogens need any support under increased acetate availability. It is pretty
obvious that we have, just from a community analysis, the presence of obligate and facilitative
anaerobes that are capable of the breakdown and fermentation of organic matter producing acetate
and hydrogen to drive methanogenesis. Warming which releases this organic matter will therefore end
up supplying these products of fermentation for methanogens
>Indeed, we wanted to mention here that the bacterial population is crucial for providing acetate for the methanogenic community. We have changed this sentence, which now reads: “Upon metagenomic sequencing we found that several bacterial species could support the methanogenic community by potentially increasing acetate availability” (line 479-480)
47. Line 459 but this is reporting on what would have been known previously in the 2018 paper
>Indeed, this is the observation of both the current and the 2018 paper, the novelty of our study is highlighted in the next sentence.
48. Table S1 Frequency is the number of occurrences of a repeating event per unit of time -do the
authors mean fractional abundances of 16S rRNA gene sequences . in the table I think a number like
0.48 -is a fractional abundance -this is not said in the table anywhere and if so what is it a
fractional abundance of? what total reads do you mean? total archaeal reads -this does not make
sense
>We have changed “Frequency data” to “Fractional abundance”. Furthermore, we’ve added the sentence “100% represents the total of 16S rRNA gene reads that were identified as archaeal reads.” To indicate the fractions are calculated from the total of archaeal 16S rRNA gene reads.
